# The Taxonomy of the Genus *Entamoeba* (Archamoebea: Endamoebidae): A Historical and Nomenclatural Review

**DOI:** 10.3390/pathogens15020213

**Published:** 2026-02-13

**Authors:** Lorena Esteban-Sánchez, Rafael Alberto Martínez-Díaz, Francisco Ponce-Gordo

**Affiliations:** 1 Department of Microbiology and Parasitology, Faculty of Pharmacy, Complutense University, Plaza Ramón y Cajal s/n, 28040 Madrid, Spain; lorees01@ucm.es; 2 Department of Preventive Medicine and Public Health and Microbiology, Faculty of Medicine, Autonomous University, C/Arzobispo Morcillo s/n, 28029 Madrid, Spain; rafael.martinez@uam.es

**Keywords:** *Entamoeba*, taxonomy, historical review, nomenclatural issues, comprehensive species inventory, taxonomic guidelines

## Abstract

Throughout history, species within the genus *Entamoeba* have been described using criteria that were not always applied consistently, resulting in an often confusing and controversial taxonomy. Several factors contributed to this situation, including the limited number of morphological characters available for taxonomic studies, overlapping host ranges, mixed infections, and a cosmopolitan distribution associated with human and animal movements. The incorporation of genetic data as diagnostic and differential criteria during the second half of the twentieth century enabled the recognition of cryptic species and the proposal of new taxa; however, significant taxonomic issues remain unresolved. This review summarizes the historical development and major controversies in the taxonomy of *Entamoeba*, from its origins in the late nineteenth century, when morphology and host association were the available criteria, to the present day, in which molecular approaches provide a more realistic view of species diversity and interspecific relationships. Based on this analysis, general principles are proposed as a pragmatic synthesis to guide future taxonomic work on *Entamoeba*, emphasising lineage-based species delimitation, the central role of molecular evidence when diagnostic morphology is lacking, the contextual value of host data, and the need for nomenclatural decisions grounded in biological evidence and historical rigour.

## 1. Introduction

The genus *Entamoeba* comprises several species of parasitic amoebae infecting vertebrates, most of them inhabiting the intestinal tract. Exceptions include a few species described from the oral cavity of various mammals, one species reported from the stomach of fish, and three species described from environmental samples; of these, a facultative free-living or host-associated lifestyle has been documented for one species (*E. moshkovskii*), whereas no evidence is currently available to assess whether a similar behaviour occurs in the other species. The vast majority of *Entamoeba* species are considered commensals; however, a small number are pathogenic. Among them, the human species *E. histolytica* has attracted the greatest research interest.

In the life cycle of intestinal *Entamoeba* species, two morphological stages are recognized: the trophozoite and the cyst (in species inhabiting the oral cavity, only trophozoites have been described). Trophozoites lack a defined shape and show few conspicuous internal structures other than the nucleus and some vacuoles; consequently, the range of morphological characters available for taxonomic purposes is essentially restricted to cell size and nuclear features. In cysts, in addition to size range and nuclear morphology, other characters can be considered, such as the presence and morphology of chromatoid bodies (ribosomes arranged in a helical pattern [1,2]), the presence of vacuoles, and the number of nuclei in mature cysts. However, chromatoid bodies frequently disappear in mature cysts, and vacuoles occur irregularly even among cysts from the same isolate. Ultimately, the set of morphological characters that can be reliably assessed in cysts is reduced to three: cyst size, the number of nuclei in mature cysts, and nuclear morphology.

Because cysts are the forms most commonly encountered in faecal samples, cyst morphology—particularly nuclear morphology—combined with host species and, in some cases, the geographical origin of the samples, has constituted the basis for the identification of most of the proposed species. Specifically, the main morphological features considered by different authors include nuclear diameter, the arrangement of peripheral chromatin, the size and appearance of the endosome, its position, and the presence and distribution of small chromatin granules and filaments between the endosome and the nuclear membrane [3]. However, in some instances, the consideration of such a wide array of characters appears to reflect more the need of investigators to provide differential criteria supporting species identifications or the proposal of new taxa than the existence of truly taxonomically informative features. As a result, the validity of some of these criteria has been questioned, both because of the influence of environmental factors and the possible occurrence of artefacts [4]. An extreme example of the failure to discriminate between artefacts and genuine characters led to the proposal of *Councilmania lafleuri* [5] (see Section 4).

In addition to morphological traits, commonly used differential criteria have included host species, possible associations with pathological conditions, and the geographical origin of the material. Nevertheless, these criteria have not always been applied consistently. In some cases, the inconsistencies went unchallenged owing to the scientific authority of the authors proposing new taxa, whereas in others (particularly those involving non-pathogenic species from animal hosts) it reflects the limited attention paid to taxa of little or no medical relevance. The outcome has been a somewhat confusing taxonomy, marked by recurrent controversies throughout the 20th century. The incorporation of immunological, physiological, biochemical, and genetic criteria, especially over the past three decades, has helped to resolve some taxonomic problems and has also generated new debates.

The aim of this review is to provide a comprehensive historical analysis of the taxonomy of the genus *Entamoeba*, tracing the successive criteria that have been applied to species recognition and delimitation from the late 19th century to the present. By revisiting original descriptions, subsequent revisions, and long-standing controversies, we seek to clarify a number of taxonomic ambiguities that have persisted in the literature, often as a consequence of inconsistent or implicitly applied criteria. In particular, this review highlights how different standards have been used to delimit species depending on host group, biological relevance, or period, and how these practices have shaped the current understanding of diversity within the genus. By compiling and critically reassessing the full spectrum of species names historically proposed for *Entamoeba*, including those later synonymized or overlooked, we provide the most complete and contextualized overview of species diversity, to date. We do not propose modifications to the taxonomic status of any species (e.g., validation as a distinct taxon or synonymizing it with other names); rather, we summarize and report the views expressed by previous authors, citing them in each case, and occasionally point out inconsistencies, insufficiently justified proposals, or uneven application of criteria. Only in Section 5 (family-group name), based on a critical reading of the relevant literature, do we discuss which term should be used in accordance with the rules of nomenclature.

Beyond its historical scope, this work aims to bridge classical taxonomy with modern molecular approaches, placing recent discoveries of cryptic diversity and genetic lineages within their appropriate conceptual and nomenclatural framework. Finally, we discuss the current status of species delimitation in *Entamoeba* and outline the main challenges that remain, particularly in relation to integrative taxonomy, the interpretation of molecular variation, and the stable application of species names. In doing so, this review seeks to offer a coherent reference framework to support future taxonomic, evolutionary, and epidemiological studies on this genus.

## 2. Early Controversies: Up to the Proposal of *Entamoeba histolytica*

Although descriptions of intestinal disorders attributed to amoebae can be recognized in the writings of ancient authors (such as the “fluxus ventris” described by Celsus and Hippocrates) the first description of a parasitic amoeba in humans was that of *Amoeba gingivalis*, reported in 1849 by G. Gros [6] and later renamed *Entamoeba gingivalis* by E. Brumpt [7]. This species inhabits the oral cavity and is generally regarded as a commensal, although it has been associated with gingival disease [8,9,10,11,12].

The first description of an intestinal amoeba is usually attributed to W. Lambl in 1860 [13], who reported amoebae in a child from Prague suffering from diarrhoea who subsequently died of enteritis. However, C. Dobell [14] argued that the amoebae described by Lambl, measuring 4–6 µm in diameter, were in fact degenerate forms of *Trichomonas hominis* (*Pentatrichomonas hominis*). Dobell instead credited T. R. Lewis [15] and D. D. Cunningham [16] with the discovery of intestinal amoebae, based on observations in Indian patients with diarrhoea, although it is possible that the symptoms were attributable to the cholera epidemic prevailing in India at that time rather than to amoebic infection.

A few years later, in 1875, the Russian physician F. Lösch proposed the name *Amoeba coli* for the “amoebae of the colon” observed in a peasant suffering from dysentery who ultimately died of pneumonia [17]. At autopsy, Lösch found numerous intestinal ulcers containing amoebae, but he did not consider the organisms to be the cause of the intestinal pathology. Instead, he attributed the disease to other aetiologies and regarded the amoebae merely as a mechanical (irritant) factor preventing ulcer healing. According to Massiutin [18], Lösch reinforced this view after examining additional patients harbouring intestinal amoebae in the absence of any associated pathology.

In retrospect, the first case studied by Lösch most likely corresponded to the species currently known as *E. histolytica*, whereas the subsequent cases probably involved *E. dispar* or *E. coli*. Lösch’s lack of taxonomic precision was later used by O. Casagrandi and P. Barbagallo [19] to (re)name the human parasitic species as *E. hominis*, and by Dobell [14] to support and justify the validation of the specific epithets introduced by F. Schaudinn in 1903 [20].

The situation became more complex when B. Grassi [21] described intestinal amoebae in the faeces of individuals without dysentery, identifying them as *Amoeba coli* sensu Lösch and considering them to be non-pathogenic. Grassi also observed cysts, but misinterpreted them as coccidia [14]. For more than three decades thereafter, published studies failed to clarify whether human intestinal amoebae were genuinely pathogenic. In some investigations of dysenteric cases, amoebae were detected and associated with the patients’ intestinal disease [22,23,24], whereas in other studies amoebae were found in asymptomatic individuals [19,25,26] or were regarded as non-pathogenic [27].

William T. Councilman and H. A. Lafleur demonstrated that dysentery may have different aetiologies, showing that amoebic dysentery is clinically and aetiologically distinct from other forms of dysentery [24]. They proposed the name *Amoeba dysenteriae* for the organism responsible for intestinal ulcerations, and *Amoeba coli* Lösch for the non-pathogenic form. However, their proposal was not supported by morphological evidence and was therefore not accepted.

The studies of H. Quincke and E. Roos [28], as well as that of Roos [29], would have been of considerable importance had they received sufficient attention, but they went largely unnoticed. These authors reported morphological differences that allowed the distinction of two different types of amoebae: those found in patients with dysentery exhibited distinctive features (such as differences in cytoplasmic appearance, nuclear morphology, the presence of phagocytosed erythrocytes, and motility) when compared with amoebae observed in non-dysenteric individuals. They also provided a very brief description of cysts, reporting diameters of 10–12 µm for amoebae associated with dysentery and 16–17 µm for those found in asymptomatic patients. However, no information was given on the number of nuclei present in either cyst type.

In essence, two main issues prevented resolution of the debate (in addition to the aforementioned lack of recognition of certain studies, partly attributable to difficulties in the dissemination and accessibility of scientific publications at the time): first, the assumption that all intestinal amoebae, whether associated with disease or not, belonged to a single species; and second, the treatment of dysentery as a disease entity rather than as a syndrome with diverse, and sometimes coexisting, aetiologies within the same patient. From a taxonomic perspective, this meant that the potential taxonomic value of morphological differences observed in trophozoites from different cases was largely disregarded, and that host species (humans) was treated as a strict criterion, under the assumption that all morphologically similar amoebae infecting the same host and occupying the same anatomical niche (the intestine) must belong to a single species.

The situation changed when the respected German zoologist F. Schaudinn published a study proposing the existence of two species of human intestinal amoebae: a commensal species (*E. coli*) and a pathogenic species causing tissue lysis, which he named *E. histolytica* [20]. Schaudinn attempted to demonstrate the innocuous or pathogenic nature of these species through self-infection experiments using material obtained from the cases he studied. Three years later, he died as a result of a liver abscess, probably caused by one of these self-infections. Schaudinn identified the octonucleate cysts of *E. coli*, but he never recognized cysts of *E. histolytica*, which he believed to form spores instead [20]. Moreover, C. Huber [30] described trophozoites and tetranucleate cysts in patients with dysentery, but following Schaudinn opinion, they were assigned to a different species, *E. tetragona* [30], a misspelling corrected to *E. tetragena* [31], one of the many junior synonyms later attributed to *E. histolytica*.

Although Schaudinn’s proposal shed light albeit with some remaining ambiguities on the debate surrounding human intestinal amoebae, it also introduced a nomenclatural inconsistency. His interpretation, which would have warranted critical scrutiny, was initially accepted largely on the basis of Schaudinn’s considerable academic authority and was subsequently retained in order to avoid confusion, particularly among clinicians. It is likely that Schaudinn was influenced by the work of O. Casagrandi and P. Barbagallo, who had proposed the generic name *Entamoeba* for human intestinal amoebae and considered *E. hominis* to be the correct name for the organism originally described by Lösch [19,26]. Assuming that Lösch’s species was non-pathogenic (supported by Lösch’s own reluctance to associate amoebae with dysentery) and that *E. hominis* was therefore a junior synonym of *Amoeba coli*, Schaudinn applied correctly the name *E. coli* to the non-pathogenic species. However, he did not take into account the name *Amoeba dysenteriae* proposed earlier by Councilman and Lafleur for a pathogenic amoeba [24]. As a consequence, the name *E. histolytica* introduced by Schaudinn was in fact a junior synonym of an earlier name. There is strong evidence that the amoeba described by Lösch as *Amoeba coli* was pathogenic and associated with dysentery. Therefore, Schaudinn should have applied the name *E. coli* to the pathogenic species rather than to the commensal one. Had Schaudinn’s proposal been revised accordingly, *E. coli* Lösch, 1875 would have been assigned to the pathogenic species, and the non-pathogenic species would have been renamed *E. hominis* Casagrandi and Barbagallo, 1895, the earliest specific name (other than *E. coli*) used for amoebae not associated with dysentery. Depending on the interpretation of Lösch’s work (pathogen or not pathogen amoeba), the senior synonym could have been either *A. coli* Lösch, 1875, or *A. dysenteriae* Councilman and Lafleur, 1891.

Proposals along these lines were indeed made [32], but they appeared nearly fifteen years after the “birth” of *E. histolytica*, at a time when a change in nomenclature would already have caused considerable confusion. To preserve Schaudinn’s proposal in accordance with the International Code of Zoological Nomenclature, Dobell suggested treating *Amoeba coli* Lösch not as a valid scientific name but as a descriptive term (“amoebae of the colon”) [14]. Under this interpretation, the first valid use of the specific epithet *coli* would be that of Grassi in 1879 [21], who applied it to non-pathogenic intestinal amoebae; consequently, *E. hominis* would be unavailable as a junior synonym of *E. coli* Grassi 1879.

This reasoning created a further difficulty: the earliest valid name for pathogenic amoebae would then have been *E. dysenteriae*. Dobell resolved this by proposing that this epithet should also be considered unavailable, arguing that Councilman and Lafleur had introduced it as a replacement for *Amoeba coli* Lösch and that it therefore constituted a junior synonym of *E. coli*. This interpretation was accepted, despite the conceptual inconsistency it concealed: Councilman and Lafleur had proposed *A. dysenteriae* specifically to replace Lösch’s name because they regarded it as non-diagnostic, and they clearly referred to a pathogenic amoeba. If *A. coli* Lösch were not a valid species name, and *E. coli* Grassi applied to a non-pathogenic species, then *E. dysenteriae* would in fact be an available name for the pathogenic taxon.

In summary, the correct names for the pathogenic and non-pathogenic species should have been either *E. coli* Lösch, 1875 and *E. hominis* Casagrandi and Barbagallo, 1895, respectively, or *E. dysenteriae* Councilman and Lafleur, 1891 and *E. coli* Grassi, 1879. Such a revision is unthinkable today, after more than a century of consistent use of the names *E. histolytica* and *E. coli* for the pathogenic and commensal human amoebae, respectively. Moreover, in accordance with the International Code of Zoological Nomenclature, prevailing usage has fixed these names, which are therefore valid in any case. One practical consequence of this historical process is that the correct authorship of *E. coli* should be attributed to Grassi, 1879, rather than to Lösch, 1875, as would otherwise have been expected.

## 3. The Genus Name: *Entamoeba* vs. *Endamoeba*

Coinciding with the period of debate and confusion at the end of the 19th century regarding the existence of one or more species of human intestinal amoebae, a parallel controversy emerged concerning the correct genus name for parasitic amoebae. Initially, parasitic amoebae were included in the genus *Amoeba* together with free-living species, until J. Leidy separated the intestinal amoebae of cockroaches (*Amoeba blattae* Bütschli, 1878) into a distinct genus, which he named *Endamoeba* [33]. The morphological characteristics of this amoeba differ markedly from those of species currently assigned to *Entamoeba*, both in trophozoites and cysts [34].

Several years after Leidy’s proposal, and apparently without knowledge of his work, Casagrandi and Barbagallo introduced the name *Entamoeba* to include the parasitic amoebae they studied in human samples [26]. The name *Entamoeba* was subsequently adopted in later studies, most notably in the influential work of Schaudinn [20]. In 1908, based on Schaudinn’s erroneous interpretation that *E. histolytica* formed spores, M. Lühe proposed that the pathogenic species should be placed in a separate genus, *Poneramoeba* [35].

Leidy’s earlier name remained largely unnoticed until E. Chatton drew attention to the possible synonymy between *Endamoeba* and *Entamoeba* [36]. Chatton and P. Lalung-Bonnaire argued that sufficient differences existed between the parasitic amoebae of cockroaches and those infecting humans to justify their placement in separate genera; moreover, considering *Entamoeba* to be too similar to *Endamoeba*, they proposed the new genus *Loschia* (in honour of Lösch) [37]. One month later, A. Alexeieff proposed the genus *Proctamoeba* to include amoebae parasitising vertebrates, including humans [38].

The growing interest in human intestinal amoebae led to the publication of numerous studies describing new genera and species, many of which were later shown to be based on contamination with free-living amoebae or on mixtures of different organisms [39]. Authors’ opinions regarding the appropriate genus name were divided, although *Entamoeba* was used most frequently (Table 1). Many of the species described during this period were subsequently regarded as synonyms; nevertheless, particular attention should be paid to the work of S. von Prowazek, who described *Entamoeba butschlii* [40], and to that of C. M. Wenyon and F. W. O’Connor, who described *Entamoeba nana* [41]. Both species were later reassigned to newly established genera, *Iodamoeba* (by Dobell in 1919 [14]) and *Endolimax* (by S. L. Brug in 1918 [42]), respectively.

In 1928, the International Commission on Zoological Nomenclature (ICZN) issued Opinion 99 [43], stating that the name *Entamoeba* should be regarded both as a homonym and a synonym of *Endamoeba*. For those authors who considered that the vertebrate species included in *Entamoeba* (with the genera *Endolimax* and *Iodamoeba* excluded from this discussion) should not be placed in the same genus as the invertebrate species, the name of the new genus could not be *Entamoeba* but rather *Loschia*, in accordance with the principle of priority. This criterion, however, was not widely accepted, and many authors argued that the two names (*Endamoeba* and *Entamoeba*) should be retained as separate genera. Indeed, the majority of newly described species continued to be assigned to *Entamoeba* (Table 1).

Following the contributions of several authoritative authors, most notably Dobell [44] and H. Kirby [45,46], who supported the retention of *Entamoeba* as a valid name, the ICZN issued a new ruling in 1954, replacing the previous one [47]. In this opinion, *Entamoeba* was accepted as a valid genus distinct from *Endamoeba*, thereby definitively resolving the issue. Adoption of *Entamoeba* was rapid, and from the 1960s onwards virtually all studies employed this name.

Surprisingly, some species originally described from invertebrates (and therefore attributable to *Endamoeba*) have been considered in recent studies as belonging to *Entamoeba* [48], and isolates from cockroaches have also been identified as *Entamoeba* [49]. However, no morphological data were provided to allow even a tentative assessment of the correctness of these identifications, and phylogenetically these isolates are placed in a separate sister clade [49]. This has led some authors to include invertebrates within the host range of *Entamoeba* (e.g., refs. [50,51]).

Spurious infections in invertebrates with access to faecal material are, however, plausible. For example, H. H. Pai and colleagues [52] detected cysts of *E. histolytica* in the intestine of cockroaches experimentally exposed to positive human faeces for up to four days post-exposure, as well as cysts of *E. histolytica*/*E. dispar* and *E. coli* in cockroaches captured in schools in Taiwan. In addition, unpublished sequences available in GenBank assigned to *E. moshkovskii* (a species originally described from wastewater [53] and known to develop in both poikilothermic and homeothermic vertebrates [54,55]) have been obtained from cockroach and beetle samples.

Considering temperature as a limiting factor for trophozoite development, these findings strongly suggest spurious parasitosis, at least for *E. histolytica*/*E. dispar* and *E. coli*. For free-living species such as *E. moshkovskii*, current data do not allow a definitive conclusion as to whether their presence in invertebrates reflects spurious infection or genuine development in the intestinal tract, similar to that observed in wastewater or environments heavily contaminated with organic matter. Even if it were ultimately demonstrated that some *Entamoeba* species (e.g., *E. moshkovskii*) can truly develop in invertebrate hosts, the clear morphological differences between *Entamoeba* and *Endamoeba* support their retention as separate genera (as is also the case for *Endolimax* and *Iodamoeba*), even if host overlap were to occur in some species.

## 4. Councilmania

The case of *Councilmania* deserves particular attention as an illustrative example of the general state of confusion that characterized the taxonomy of intestinal amoebae and the identification of species infecting humans in the early 20th century, as well as of the influence of technical limitations and interpretative errors on the use of morphological criteria for protozoan species delimitation.

In their 1921 study, C. A. Kofoid and O. Swezy considered the existence of several species that could be present in human faeces, namely *Endamoeba dysenteriae* (as discussed above, a synonym of *E. histolytica*), *Endamoeba coli*, *Endolimax nana*, *D. fragilis* (which is not an amoeba but a flagellate), and *E. muris* [5]. They employed different genus names for *E. dysenteriae* (*E. histolytica*) and *E. coli*, and for *E. muris*. Within *E. coli*, they distinguished two forms: in addition to the typical species, they identified a second entity, which they named *Councilmania lafleuri*.

This new species was characterized by its resistance to haematoxylin staining, the presence of eight chromosomes (as opposed to six in *E. coli*), and, most notably, by cyst features such as a predominantly asymmetric shape, particular patterns of chromatin distribution within the nuclei, and the supposed budding and escape of amoebulae from cysts. Shortly after this proposal, several authors [56,57] questioned the validity of *C. lafleuri*, reporting that they were unable to reproduce the differences described by Kofoid and Swezy, whereas others accepted the new species [58]. Kofoid and his collaborators nevertheless maintained their proposal. They expanded the host range of *C. lafleuri* to include rats [59], added new species from rodents (*C. decumani* and *C. muris* [60]), and transferred additional taxa to the genus. Thus, *E. tenuis* Kuenen and Swellengrebel, 1917 (a synonym of *E. histolytica* or, more likely, of *E. hartmanni*) was renamed *C. tenuis*, and a new human species, *C. dissimilis*, was erected [61].

Kofoid and colleagues responded to critical assessments [62,63], but a substantial controversy regarding the validity of *C. lafleuri* persisted. In the following years, arguments both supporting and rejecting its validity were presented by T. H. T. Wight and L. H. Prince [64] and R. J. Pickard [65,66]. As an intermediate solution, it was even proposed to rename the species as *Endamoeba lafleuri* [67].

The controversy was finally resolved by R. M. Stabler in 1932 [68], who conducted a detailed study of a patient infected with *E. coli*, with *Blastocystis hominis* as the only potentially confounding organism. Stabler demonstrated that fixation type and temperature had a clear influence on the subsequent appearance of cyst budding, and that all the purported diagnostic morphological differences of *C. lafleuri* had either already been described in the literature for *E. coli* or could be observed in his own material; then, it was a synonym of *E. coli*. Following this study, no further discussion on the validity of *C. lafleuri* (or the genus *Councilmania*) ensued.

## 5. The Family Name: *Endamoebidae* vs. *Entamoebidae*

A further issue arising from the debate on the validity of the generic name *Entamoeba* concerns the correct family name. According to Article 29 of the International Code of Zoological Nomenclature (the Code) [69], family-group names must be formed from the name of the type genus, and Article 64 states that any genus considered valid may be designated as the type genus, not necessarily the oldest one.

It is important to note that, as discussed above, the name *Endamoeba* itself was never in dispute; the debate focused exclusively on whether *Entamoeba* should be regarded as a valid and independent genus. Both before and after the definitive resolution of the generic nomenclature in 1954, the family name Endamoebidae Calkins, 1926 was widely used in classic handbooks to include both *Endamoeba* and *Entamoeba* [70,71,72,73,74,75], as well as in more recent taxonomic publications and reviews [48,76,77,78,79]. Occasionally, some authors have used Endamoebidae for the family while designating *Entamoeba* as the type genus [80,81], a contradictory practice that does not conform to the provisions of the Code. However, since the adoption of the name Entamoebidae Chatton, 1925 by Thomas Cavalier-Smith in 1991 [82], its use has increased steadily. This name has been employed in global taxonomic revisions [83,84] as well as in numerous focused studies [85,86,87,88,89,90,91], and it is the family name currently used, at the time of writing, in databases such as GenBank.

During the 19th century, intestinal amoebae were generally included in the family Amoebidae, a classification usually attributed to C. G. Ehrenberg [92]. The vast majority of studies published in the early 20th century addressing taxonomy, species validity, or the *Entamoeba* vs. *Endamoeba* debate made no reference to the family level and did not designate a type genus. The family-group name Entamoebidae was introduced by Chatton [93]. However, when establishing this family, Chatton wrote:

“6° Entamoebidae, dont certaines, comme l’*Entamoeba blattae*, ne se distinguent guère des précédentes. Synénergides comme elles, à gamètes non flagellés comme ceux d’*Amoeba flava*. Mais l’*Entamoeba blattae* n’est certainement pas la forme la plus centrale du groupe. Les Entamibes des Vertébrés, autant qu’on en peut juger par le peu qu’on en connaît, paraissent s’en écarter notablement. Leur cycle est d’ailleurs inconnu. Cependant la schizogonie d’*E. ranarum* (Collin, 1913) ou celle d’*Entamoeba coli* (Mathis et Mercier, 1917), à défaut de la gamétogenèse, mettent bien en évidence la condition synénergide de ces Amibes.” [93] (p. 50).

Although apparently derived from the generic name *Entamoeba*, Chatton neither designated a type genus nor applied the family name in a manner consistent with the nomenclatural rules in force at the time. The validity of *Endamoeba* itself was never under discussion; Chatton’s reference to *Entamoeba blattae* is a misidentification of *Endamoeba blattae*, as no justification for this reassignment is provided in the original work, and the same confusion is already evident in one of his earlier publications [36] (p. 282):

“Ce sont d’une part les Amibes parasites que l’on a réunies, provisoirement sans doute, mais bien artificiellement dans le genre *Entamoeba* Leidy (1879).”

Under the Règles internationales de la Nomenclature zoologique (the Règles) [94], which governed zoological nomenclature at the time, Chatton’s family-group name Entamoebidae was not validly established, as he neither designated a type genus nor applied the name in accordance with the Règles. According to Article 31 of the Règles, a name based on an erroneous identification cannot be retained for the misidentified species and therefore cannot serve as a valid foundation for a family-group name. Furthermore, Articles 4 and 29–30 required every family name to be explicitly linked to an existing and clearly defined genus name, a condition that is not met in Chatton’s text.

If Chatton’s use of *Entamoeba blattae* were interpreted merely as an orthographic error (that is, an inadvertent misspelling of *Endamoeba*) then, under Article 25 of the *Règles*, the corresponding family name could be corrected to *Endamoebidae*. The current Code similarly allows the correction of “incorrect original spellings” when these result from evident lapsus or typographical errors (Arts. 32.4–32.5). However, if Chatton’s error is interpreted not as orthographic but as conceptual (affecting the taxonomic application of the genus name rather than its spelling) then it falls under the provisions of Article 31 of the Règles and Articles 67.13–67.14 of the Code, which state that names based on misidentified taxa cannot be retained or corrected by emendation. In this latter case, *Entamoebidae* cannot be regarded as a correctable spelling of Endamoebidae, but must instead be considered an invalid name established on an erroneous taxonomic concept. Under both the Règles and the modern Code, a misidentified species cannot validly fix a type genus (Arts. 61.1.3, 63.2), and a family-group name lacking a properly fixed type is therefore of uncertain application.

In 1926, G. N. Calkins introduced the family-group name Endamoebidae, stating:

“Family 3. Endamoebidae: these are parasitic amoebae … The genus generally recognized is represented by a vast number of species with ill-defined diagnostic characters (genus: *Endamoeba*), while other genera (e.g., *Endolimax*? *Councilmania*, etc.) are forms about which the taxonomic position is still in dispute” [95] (p. 338).

In this case, *Endamoeba* is explicitly used as the type genus, thereby fulfilling the requirements of both the Règles (Art. 4) and the modern Code (Arts. 13.1, 63.2). Calkins’ action therefore provided the first validly typified family-group name for these amoebae, independently of the temporary confusion surrounding the relationship between *Endamoeba* and *Entamoeba*. Although Endamoebidae was formally published one year after Entamoebidae, it entered prevailing usage shortly after its introduction and well before 1961. In accordance with Article 40.2 of the Code, even if Entamoebidae were considered acceptable, a family-group name replaced before 1961 that has achieved prevailing usage is to be maintained.

Therefore, given the invalidity of Entamoebidae under both the historical Règles and the modern Code, and the consistent and widespread use of Endamoebidae throughout the 20th century, the reinstatement of Entamoebidae Chatton, 1925 by Cavalier-Smith [82] cannot be supported. Depending on how Chatton’s name is interpreted (either as an incorrect spelling or as a taxonomically inconsistent usage) the correct and valid family-group name for amoebae including *Endamoeba* and *Entamoeba* would therefore be either Endamoebidae Chatton, 1925 emend. Calkins, 1926, or Endamoebidae Calkins, 1926 (=1925 by priority).

In our view, Chatton’s usage can be interpreted as reflecting a taxonomically inconsistent concept (treating *Entamoeba* as the sole genus encompassing intestinal amoebae of both vertebrates and invertebrates) rather than a simple orthographic lapse, which might reasonably occur once but be unlikely to be repeated across different publications. Accordingly, the second option, Endamoebidae Calkins, 1926, may be considered a plausible choice for the correct and valid family-group name.

## 6. Criteria for Species Differentiation in *Entamoeba*: *E. histolytica*, *E. coli* and Morphologically Compatible Species

Schaudinn validated the distinction between *E. histolytica* and *E. coli* on the basis of a biological criterion, namely the ability or inability to cause disease in the infected host [20]. Taking into account the data provided by H. Quincke and E. Roos [28] and by Roos [29], several morphological and biological criteria were already available at that time that could have allowed the identification of both species. However, differentiation between *E. coli* and *E. histolytica* proved difficult, because Schaudinn’s description of *E. histolytica* was based exclusively on trophozoites, the observations of Quincke and Roos were largely ignored, and the tetranucleate cysts described by Huber in 1906 [30] were, as noted above, assigned to a different species. It was not until the work of E. L. Walker in 1911 [96] and Walker and A. W. Sellards in 1913 [97] that it was clearly established that *E. coli* forms octonucleate cysts, whereas *E. histolytica* forms tetranucleate cysts. This clarification led to the recognition of *E. tetragena* (and several other described taxa) as synonyms of *E. histolytica*.

One consequence of this period of uncertainty (either due to the lack of well-defined diagnostic characters or to their incomplete acceptance) was that early 20th studies on human intestinal amoebae resulted in the proliferation of new species names. The criteria used by different authors to propose new species were based mainly on minor morphological differences, on the geographical origin of the cases, or on alternative nomenclatural solutions intended to address the *Entamoeba*–*Endamoeba* problem. The extent of the prevailing confusion is illustrated by the number of species described from humans during the first quarter of the 20th century (Table 2).

To this list, *E. polecki* should be added, a uninucleate cyst-forming species described by S. von Prowazek in 1912 [98]. The validity of this species was subsequently debated (see Section 7.3), as some authors considered the original description insufficient and questioned whether the organism described could even be confidently identified as an amoeba.

**Table 2:** Human intestinal amoeba species proposed in the first quarter of the 20th century.

Current Accepted Species	Original Species Name
*Entamoeba histolytica*	
	*E. tetragena* Huber 1906 (misspelled as *E. tetragona* in the original description)
	*E. dysenteriae* (Councilman and Lafleur 1891) Craig 1905
	*E. africana* Hartmann 1907
	*E. schaudinni* Lesage 1908 *
	*E. minuta* Elmassian 1909
	*E. nipponica* Koidzumi 1909
	*E. minutissima* Brug 1917
	*E. tenuis* Kuenen and Swellengrebel 1917
	*E. paradysenteria* Chaterjee 1920
	*Caudamoeba sinensis* Faust 1923
	*Karyamoebina falcata* Kofoid and Swezy 1924
	*Councilmania dissimilis* Kofoid 1928
*Entamoeba hartmanni*	
	*E. hartmanni* Prowazek 1912
	*E. minuta* Woodcock and Penfold 1916 **
*Entamoeba dispar*	
	*E. dispar* Brumpt 1925
*Entamoeba coli*	
	*Amoeba hominis* Walker 1908
	*E. loeschi* Lesage 1908 *
	*E. tropicalis* Lesage 1908 *
	*E. williamsi* Prowazek 1911
	*E. brasiliensis* Aragao 1912
	*E. intestinivulgaris* Aragao 1917
	*Councilmania lafleuri* Kofoid and Swezy 1921

(*) Several authors attributed these species to A. Lesage [99], however Lesage did not indicate the names as n.sp. neither indicated if they were previously described by other authors. (**) The name used by H. M. Woodcock and W. J. Penfold in 1936 [100] was previously used by M. Elmassian in 1909 [101], but Woodcock and Penfold though it was a different race or species (most likely it corresponded to *E. hartmanni*, according to their description).

### 6.1. Entamoeba Histolytica and Related Species in Humans and Non-Human Primates

Leaving aside proposals related to genus nomenclature, human species whose distinguishing characters were based primarily on geographical origin were regarded by Dobell and Wenyon as synonyms of the species then recognized in humans [14,102], namely *E. gingivalis*, *E. coli*, *E. histolytica*, *Endolimax nana*, *Iodamoeba butschlii*, and *D. fragilis* (now known to be a flagellate rather than an amoeba).

In 1917, Wenyon and F. W. O’Connor [41] reported from Egypt what they referred to as different “strains” of the parasite, one of which produced smaller cysts (7–10 µm) than usual, while being otherwise indistinguishable. This proposal (the existence of races or strains) was accepted by Dobell in his 1919 monograph [14], who considered that the descriptions of *E. hartmanni* [40], *E. tenuis* [103], *E. minutissima* [104], and *E. minuta* [100] corresponded to a small-cyst-producing race of *E. histolytica*.

Closely linked to the concept of races of *E. histolytica*, a new problem soon emerged from the growing body of studies on human intestinal amoebiasis: why did this species not always cause dysentery? Prior to the work of Councilman and Lafleur [24], dysentery was generally assumed to be caused exclusively by intestinal amoebae. However, it is likely that in some patients dysentery was amoebic in origin, whereas in others it was bacterial; nevertheless, because these patients were also infected with amoebae detectable in faecal samples, the disease was attributed to amoebae rather than to bacteria. Councilman and Lafleur distinguished amoebic dysentery from dysentery of other causes, introduced the term amoebic dysentery, and established its association with liver abscesses [24]. Almost a decade after Schaudinn’s description of *E. histolytica*, E. L. Walker [96] and Walker and A. W. Sellards [97] confirmed that only this species, and not other intestinal amoebae, was responsible for amoebic dysentery. Nevertheless, no explanation was available as to why *E. histolytica* did not invariably cause disease. Epidemiological data showed that many infected individuals were asymptomatic, and that only a relatively small proportion of cases (approximately 10–20%) developed clinical symptoms. Indeed, in the experimental study of Walker and Sellards, conducted with human volunteers, the results demonstrated that it was not possible to predict whether dysentery would develop or not.

In an attempt to explain the observed epidemiological patterns, two main lines of interpretation were initially proposed. On the one hand, W. A. Kuenen and N. H. Swellengrebel [105] suggested that *E. histolytica* possesses pathogenic potential but usually behaves as a commensal, causing tissue damage (such as dysentery or liver abscesses) only in a subset of infections. On the other hand, Dobell [14] argued that *E. histolytica* is intrinsically pathogenic and always produces intestinal ulcerations, but that in most cases a healthy host is able to counteract the damage, resulting in asymptomatic infections; dysentery would occur only when this balance fails.

These two views were later termed “commensalists” and “Prometheans” by R. Elsdon-Dew [106], the latter alluding to the myth of Prometheus, whose wounds regenerated continuously. Continental European researchers, particularly German authors, largely adopted the commensalist view, whereas British and American investigators tended to support the Promethean interpretation. Within the commensalist framework, the concept of races of *E. histolytica* was incorporated, with the small-sized form (minuta) regarded as non-pathogenic and the large-sized form (magna) considered variably pathogenic. In contrast, Prometheans interpreted these forms merely as size variants with identical biological behaviour.

The commensalist view prevailed until the late 1970s, when the existence of a morphologically indistinguishable but non-pathogenic species (*E. dispar*) was demonstrated, whereas the Promethean view declined by the early 1950s. The development of an in vitro culture medium for *E. histolytica* by W. D. Boeck and J. Drbohlav in 1925 [107] enabled Dobell to conduct extensive comparative studies on human and primate amoebae, published between 1928 and 1952, ultimately leading him to accept that *E. histolytica* could indeed behave as a commensal.

Building on the commensalist line, Brumpt [108,109] proposed that the so-called minuta form represented a distinct species, adopting the name *E. hartmanni* proposed by von Prowazek in 1912 [40], and that the magna form comprised two separate species: a non-pathogenic one (*E. dispar*) and a pathogenic one, for which he proposed the name *E. dysenteriae* instead of *E. histolytica*. Brumpt’s proposal was only partially accepted, and mainly with respect to the minuta form, giving rise to renewed disagreement.

English-speaking authors, following Dobell’s views (e.g., Wenyon [102]), regarded the minuta form as a mere variant of *E. histolytica*, an interpretation later known as the “unicyst” theory. Owing to the influence of Dobell and Wenyon, this view was widely adopted in the English-language literature, notably through authoritative textbooks such as “Clinical Parasitology” by C. F. Craig and E. C. Faust [110]. In contrast, a “pluralist” approach was defended by several non-Anglophone authors [111,112], who considered the consistent size differences in trophozoites and cysts, together with their lack of association with disease, sufficient to validate *E. hartmanni* as a distinct species. An intermediate position was proposed by C. A. Hoare [113], who recognized a single species (*E. histolytica*) comprising two subspecies, *E. histolytica histolytica* and *E. histolytica hartmanni*.

The size difference observed between cysts of the minuta and magna races was clear [111] and supported the recognition of the minuta form as a distinct species, *E. hartmanni* [114,115]. In contrast, trophozoite size could not be reliably used for comparisons among studies, as it is strongly influenced by the nutritional richness of the environment, both in vitro and in vivo [116]. Indeed, as early as the 1960s it was reported that *E. histolytica* trophozoites recovered from liver abscesses were larger than those obtained from the intestinal lumen [4]. The application of immunological criteria [117] further supported the morphological differentiation, and from the 1960s onwards the minuta race was generally accepted as a valid species, *E. hartmanni* [4,106,118].

By contrast, no morphological evidence was available to support the distinction between the two species proposed for the magna race (*E. dispar* and *E. histolytica* [=*E. dysenteriae*]). Consequently, *E. dispar* was initially regarded by most authors as a synonym of *E. histolytica* (see [109] (pp. 115–124)). Although this interpretation was later shown to be incorrect, it was arguably the most reasonable conclusion given the data available at the time. One could invoke the precedent of the earlier confusion between *E. coli* and *E. histolytica*, which had initially been considered a single species; however, that controversy was ultimately resolved by the recognition of clear morphological differences (particularly in nucleus morphology and number of nuclei in mature cysts) that corroborated biological differences in pathogenicity. Such morphological distinctions were absent in the case of the magna forms. In practice, priority was therefore given in this case to morphological criteria (the absence of detectable morphological differences in organisms infecting the same host species) over biological criteria such as pathogenicity. This preference was likely driven by the fact that the only diagnostic tools available at the time were morphology-based: if two putatively distinct parasites could not be distinguished morphologically, the simplest solution was to treat them as variants (strains, races, or lineages) of a single species. Notably, this criterion was not applied consistently in the description of species infecting animal hosts (see Section 7).

Unlike other points of taxonomic disagreement, the proposal that *E. dispar* represented a distinct species was largely dismissed, and no substantive debate followed Brumpt’s proposal for the next five decades. Although no mechanisms were identified to explain the apparent shift between pathogenic and non-pathogenic behaviour, there were likewise no methodological tools available to demonstrate the alternative hypothesis of two distinct species.

The introduction of additional identification and differentiation methods based on molecular characters (such as antigens, isoenzymes, and DNA) provided more refined analytical tools for the discrimination of cryptic species. In the case of *E. histolytica* and its pathogenic and non-pathogenic forms, a major turning point was the work of P. G. Sargeaunt and colleagues [119], who demonstrated clear differences in isoenzyme profiles (zymodemes) between invasive and non-invasive forms of *E. histolytica*. This finding proved to be of major relevance for clinical diagnosis [120].

The development of new in vitro culture media for *Entamoeba* [121,122] facilitated the isolation and long-term maintenance of strains. Under these conditions, changes in zymodeme profiles were occasionally observed, sometimes from virulent to non-virulent types [123], but more frequently from non-virulent to virulent ones [124,125,126,127]. Concomitant antigenic and genetic changes were also reported [128]. These observations were initially interpreted as evidence that a single species was capable, under certain unidentified conditions, of switching between non-pathogenic and pathogenic zymodemes. However, an alternative explanation was proposed [129]: zymodeme changes resulted from culture contamination. Under this view, apparent zymodeme conversion would reflect the replacement of the original strain by another introduced through inadequate laboratory practices and favoured by culture conditions. The accumulation of consistent differences based on biochemical and genetic criteria ultimately demonstrated that pathogenic and non-pathogenic zymodemes corresponded to distinct species. As a result, Brumpt’s original proposal was revisited, and the name *E. dispar* was reinstated for non-pathogenic zymodemes, while *E. histolytica* was retained for pathogenic ones [130,131,132].

The incorporation of biochemical and genetic data as differential and diagnostic criteria, developed during the second half of the 20th century, made it possible to distinguish several cryptic species previously masked under *E. histolytica*. In addition to *E. dispar*, these approaches demonstrated that humans may also be infected by three other morphologically indistinguishable species: *E. moshkovskii*, *E. bangladeshi*, and *E. nuttalli*.

*Entamoeba moshkovskii* is one of the few species within the genus that is primarily free-living, although it also exhibits infective capacity. It was originally described from wastewater in Moscow by L. E. Tshalaia in 1941 [53] and was subsequently reported from other geographic regions. Despite being morphologically indistinguishable from *E. histolytica*, its occurrence in environmental samples rather than in hosts justified its recognition as a distinct species, a taxonomic status that has never been questioned and has been confirmed by molecular analyses [133,134].

The habitats in which *E. moshkovskii* is most frequently detected (wastewater and organic-rich sediments) closely resemble the intestinal environment of animals. For this reason, R. A. Neal [4], despite unsuccessful experimental infection attempts in amphibians and mammals (including humans), did not exclude the possibility that *E. moshkovskii* could be parasitic in some, as yet unidentified, host, with the ability to survive outside the host in intestine-like environments.

In 1961, a case of human amoebiasis with diarrhoea was reported from Laredo (Texas), involving an amoeba initially identified as *E. histolytica* but displaying unusual traits, such as growth at room temperature and osmotolerance [135]. These characteristics closely resembled those of *E. moshkovskii*; however, because the isolate originated from a human host, it was designated the “Laredo strain” of *E. histolytica*. Subsequent molecular analyses unequivocally identified this strain as *E. moshkovskii* [136]. Since then, *E. moshkovskii* has been repeatedly detected in human infections [137,138,139,140,141,142,143,144,145,146,147], as well as in pigs, non-human primates (NHPs), and snakes [54,55,148]. It remains unclear whether *E. moshkovskii* is primarily an intestinal species capable of surviving in external environments rich in organic matter, or a free-living amoeba that can colonize the intestinal tract of poikilothermic and homeothermic hosts and produce transient infections.

In parallel with the description of *Entamoeba* species in humans and the debate over their validity, several species were also described from NHPs, often being related to human taxa and thereby raising similar questions regarding their taxonomic status. The first *Entamoeba* species described from NHPs was *E. nuttalli* [149], based on trophozoites (some containing erythrocytes) obtained from a liver abscess in *Macaca sinica* (=*Macacus pileatus*). Subsequently, several species morphologically similar to *E. histolytica* and associated with pathology were described from monkeys (*Macaca*, *Ateles*, *Cercopithecus*), including *E.* [=*Loschia*] *sinici* [37], *E.* [=*Loschia*] *duboscqi* [150], *E. cercopitheci* [151], *E.* [=*Ameba*] *ateles* [152,153], and *E. cynomolgi* [154]. Other species morphologically similar to *E. coli* and apparently non-pathogenic were also described (see below).

Some authors, however, considered that the pathogenic species infecting macaques was *E. histolytica* itself [155], and experimental infections in primates further supported the idea that the same species (*E. histolytica* and *E. coli*) infected both humans and NHPs [156]. Although host species was regarded by some authors as a valid differential criterion [154], the lack of consistent morphological differences led most investigators to consider *E. histolytica*–like species described from monkeys as synonyms of *E. nuttalli*, and *E. nuttalli* itself as a possible synonym of *E. histolytica* [4,14,102,156,157].

*Entamoeba chattoni*, described from macaques [158], was initially considered an *E. histolytica*–type organism [102] until H. Salis demonstrated that it formed only uninucleate cysts, thereby establishing it as a distinct species [159]. In the case of *E. cynomolgi*, the cyst sizes reported by Brug in 1923 [154] were <10 µm and most likely corresponded to *E. hartmanni*, a species not accepted as distinct until the 1960s. Molecular studies have since identified *E. hartmanni* in NHPs, including monkeys and apes [148,160,161].

No further taxonomic debate regarding *E. histolytica*–like isolates from NHPs occurred for much of the 20th century, and morphologically compatible findings in primates were generally identified as *E. histolytica* (or *E. histolytica*/*E. dispar*) until the early 21st century [162]. In 2007, H. Tachibana and colleagues resurrected the name *E. nuttalli* for a virulent rhesus monkey isolate that was genetically distinct from *E. histolytica* [163]. These authors did not exclude the possibility that this species could also infect humans, a hypothesis subsequently confirmed by genetic analyses [164,165].

Tricia L. Royer and colleagues described *E. bangladeshi* from human samples collected in Bangladesh [166], making it one of the most recently recognized species in the genus. Previous genetic surveys in the local population had identified *E. histolytica*, *E. dispar*, and *E. moshkovskii*, but a subset of positive samples failed to amplify with the molecular markers employed [138]. *Entamoeba bangladeshi* can grow in culture at room temperature, as do *E. moshkovskii* and *E. ecuadoriensis*, and is morphologically indistinguishable from *E. histolytica* and *E. moshkovskii* [138], and therefore also from *E. ecuadoriensis*, which was described as morphologically identical to *E. moshkovskii* [167]. In contrast to the criterion applied to *E. coli* (which has been maintained as a single species comprising morphologically indistinguishable but genetically distinguishable variants, see below), *E. bangladeshi* was designated as a distinct species despite the absence of diagnostic morphological characters, on the basis of clear genetic differences from *E. histolytica*, *E. dispar*, *E. moshkovskii*, and *E. ecuadoriensis*.

In summary, the taxonomic treatment of *E. histolytica* and related species in humans and non-human primates was initially governed by a conservative principle whereby the absence of consistent morphological differences was taken as evidence against species separation. Under this framework, variability in pathogenicity and host association was interpreted as intraspecific variation, leading to the long-standing inclusion of forms now recognized as distinct species within *E. histolytica*. The application of the same criterion to primate isolates further reinforced this view. The subsequent incorporation of biochemical and genetic data fundamentally altered this perspective, demonstrating that morphologically indistinguishable lineages could represent independently evolving species and leading to a reassessment of species boundaries within this group.

### 6.2. Entamoeba coli and Related Species in Humans and Non-Human Primates

Studies on *E. coli* and morphologically compatible isolates have been comparatively fewer and less influential than those focusing on *E. histolytica*, and the associated debate has been much less intense, with the notable exception of *Councilmania*. Several species morphologically similar to *E. coli* were described from humans during the early 20th century (Table 2), but they were regarded as synonyms by Dobell [14], Wenyon [102], and, in the case of *C. lafleuri*, by Stabler [68]. Dobell’s authoritative opinion was subsequently followed, and the human amoeba forming octonucleate cysts has consistently been identified as *E. coli* [73].

In NHPs, three species with *E. coli*–like morphology were described: *E. pitheci* from orangutans [40], *E.* (=*Loschia*) *legeri* from macaques [150], and *E. multinucleata* from orangutans [168]. Their taxonomic validity was also questioned by Dobell and Wenyon [14,102,169]. Based on his own studies and those of other authors, Dobell [169] concluded that the octonucleate cyst-forming amoeba infecting primates was either *E. coli* or another species that should be named *E. pitheci*; however, because no reliable differences could be identified between *E. pitheci* and *E. coli* other than host species, he treated the former as a synonym of *E. coli*, applying the same pragmatic criterion used during the first half of the 20th century in the *E. histolytica*–*E. dispar* case.

Because *E. coli* is non-pathogenic, it has not been a major focus of research, and there has been little debate regarding the taxonomic status of new isolates from humans or primates, which have routinely been identified as *E. coli*. By the late 20th century, intraspecific variants were recognized in human isolates (defined as zymodemes [170], ribodemes [133], or genotypes [134,171]). Charles G. Clark and L. Diamond [133], following the criteria of Dobell and Wenyon, concluded that in the absence of morphological or other diagnostic differences allowing clear discrimination, it was preferable to retain these variants within a single species, *E. coli*. This represents a pragmatic approach, similar to that historically applied to *E. dispar*, but the contrast in taxonomic treatment relative to *E. histolytica* and its morphologically similar relatives is evident. More recently, H. M. Elsheikha and colleagues [162] suggested, on the basis of genetic analyses, that *E. coli* may comprise cryptic species; however, no formal taxonomic separation was proposed, as no differences other than sequence divergence are currently known. Thus, despite the recognition of genetic diversity within *E. coli*, species delimitation has remained anchored to a conservative framework, in which sequence divergence alone has not been considered sufficient to justify taxonomic separation.

## 7. Identification and Differentiation Criteria Applied to the Remaining Species of the Genus *Entamoeba*

At the beginning of the 20th century, amid the intense debate over the existence of one or multiple species of human intestinal amoebae, their pathogenic or non-pathogenic nature, and even the appropriate generic name to be applied (*Entamoeba* vs. *Endamoeba*), studies on intestinal amoebae of animals were also initiated. These investigations led to the description of new species and, in some cases, to further debates regarding their taxonomic validity.

Within *Entamoeba*, species characteristically form mature cysts containing a specific number of nuclei: one, four, or eight. Hoare [172] proposed grouping *Entamoeba* species into morphological categories based on the number of nuclei in mature cysts. Norman D. Levine subsequently designated these groups using the name of a representative species from each category, referring to them as the histolytica group (tetranucleate cysts), the coli group (octonucleate cysts), the bovis group (uninucleate cysts), the gingivalis group (no known cyst stage), and an unnamed group for the insufficiently known species [73]. For ease of presentation, this system is adopted in the following sections. However, although the proposal of morphological groups initially appeared attractive and was supported by early genetic data [133,134], subsequent analyses have demonstrated that these groups are polyphyletic [89,162,173]. Moreover, as discussed in the following sections, there is considerable variability in host range and ecological niche (intestinal tract, oral cavity, environmental samples) among the species included within each morphological group.

Overall, the taxonomic treatment of *Entamoeba* species infecting non-human hosts has been shaped by a heterogeneous and context-dependent application of diagnostic criteria. Across different morphological groups, species delimitation has variously relied on cyst morphology, host association, geographic origin, or minor trophozoite features, often in the absence of comparative analyses. In contrast to the conservative approach historically applied to human and primate isolates, host specificity was frequently invoked as a decisive criterion in animal hosts, leading in some groups to taxonomic inflation rather than synonymization. The later incorporation of molecular data has revealed substantial hidden diversity but has not yet been systematically reconciled with historically described species, leaving the taxonomic status of many animal-associated lineages unresolved.

### 7.1. Entamoeba Species Forming Mature Tetranucleate Cysts (Histolytica Group)

Studies on amoebae of the histolytica group were soon extended to other mammalian hosts, particularly NHPs, with morphology and host species being used as the main taxonomic criteria. The taxonomic difficulties associated with these species have already been outlined above (see Section 6). In mammals other than primates, amoebae assigned to the histolytica group have also been described in horses and dogs. The species described in this group are indicated in Table 3.

Harold B. Fantham studied amoebae detected in two horses with diarrhoea in South Africa and described the species *E. equi* [175]. The only characters he considered to differ from *E. histolytica* were trophozoite length (larger in *E. equi*) and nuclear morphology (elongated rather than rounded); the cysts (of which Fantham reported only three) were described as identical to those of *E. histolytica*. No further records of this species were reported for almost 80 years, until Clark and colleagues detected amoebic trophozoites in cultures derived from horse faecal samples [196]; no cysts were observed and no detailed morphological data were provided to assess whether the trophozoites corresponded to Fantham’s description because the observations were based on in vitro cultures and then trophozoite size was considered potentially uninformative [197]. Nevertheless, these authors interpreted the isolates as *E. equi* because they considered it to be the only species previously described from horses (it appears that they were unaware of the description of *E. gedoeltsii* [198]), and because molecular analyses showed that the isolate clustered phylogenetically with species of the histolytica group. Other authors have argued that, in the absence of evidence demonstrating the formation of tetranucleate cysts and clear morphological compatibility with the original description of *E. equi*, it would have been preferable to identify the isolate as *Entamoeba* sp. [199], since phylogenetic affinity alone is insufficient to assign an isolate to a particular morphological group (for example, *E. bovis* forms uninucleate cysts but is phylogenetically included within the histolytica group [51,162,173,200,201,202,203,204,205,206,207,208,209,210,211]). More recently, a second ribosomal lineage (designated RL9), more closely related to a bovine isolate than to *E. equi*, has been identified [202].

In canids, *E. caudata* was described by A. Carini and E. Reichenow in 1949 [176] from a case of dysentery in a dog, but it was regarded as a synonym of *E. histolytica* [172]. Immunological analyses of canine isolates [212] and subsequent genetic studies [213] have identified *E. histolytica*, *E. dispar*, and *E. moshkovskii*; thus, there is no evidence that dogs are infected by an *Entamoeba* species distinct from these taxa.

In birds, two species assigned to the histolytica group have been described: *E. lagopodis* and *E. anatis*. Both were described by Fantham in South Africa [177,178]. *Entamoeba lagopodis* was reported from a grouse and, according to the author, was not associated with pathology [178]. *Entamoeba anatis* was described from a duck that had died from acute enteritis, and the author had access only to faecal samples, in which he observed trophozoites and cysts resembling those of *E. histolytica* [177]. In both instances, Fantham considered the organisms to represent new species without performing detailed comparative analyses. Subsequently, E. R. Hegner in 1929 [214], in experimental infections of chickens using intestinal contents from ducks, observed amoebic trophozoites that he considered potentially attributable to *E. anatis*; however, because no cysts were detected, he refrained from making a definitive identification.

Data on tetranucleate cyst-forming *Entamoeba* species infecting amphibians are scarce. The species *Amoeba ranarum* was originally described from frogs by Grassi in 1879 [21]. In 1907, Hartmann [191] described *E. ranae*; however, because of its close morphological similarity to Grassi’s species and in accordance with the principle of priority, Dobell [215] renamed it *E. ranarum* and subsequently identified toads as natural hosts [216].

Several decades later, E. A. Lobeck [192] described *E. pyrrhogaster* from Japanese salamanders (*Cynops* [=*Triturus*] *pyrrhogaster*) imported into the United States for research purposes. The only distinguishing character proposed was host species (salamanders vs. frogs). Lobeck justified this criterion by reporting negative results from experimental infections of frogs with organisms obtained from salamanders; however, these experiments were limited, as only two frogs were inoculated. In contrast, J. Cairns [193], in a study of cross-infections among anurans and urodeles (and other vertebrate groups), examined Japanese salamanders harbouring amoebae identified as *E. ranarum* and successfully transmitted the infection from salamanders to frogs.

The first molecular data for *E. ranarum* were provided by Clark and Diamond [133] and Silberman et al. [134], based on isolates from the African bullfrog (*Pyxicephalus adspersus*). More recently, outbreaks in wild cane toads (*Rhinella marina*) in Australia [217] and in captive Cranwell’s horned frogs (*Ceratophrys cranwelli*) in the United States [218] revealed genetically distinct isolates associated with pathology, designated *Entamoeba* sp. CT1 (OTU_12), as well as a possible third species, *Entamoeba* sp. OTU_119 [217]. However, no information is available on the cyst morphology of these isolates, precluding confirmation that they belong to the tetranucleate morphological group. As stated above, genetic similarity does not necessarily imply morphological similarity, and pathogenicity alone is not indicative of cyst nuclear number, as *E. ilowaiskii* is a pathogenic, uninucleate cyst-forming species infecting amphibians [219].

After mammals, reptiles constitute the vertebrate group in which the highest number of *Entamoeba* species of the histolytica group have been described, including species from lizards, snakes, and especially turtles. Some of these taxa remain frequently used in current studies, whereas others appear to have been largely overlooked.

The first amoeba described from reptiles was *Amoeba lacertae*, reported by M. Hartmann and S. von Prowazek [180] from European lacertids. Presumably the same organism was later examined by K. Nägler [220] and Dobell [221], and based on their descriptions, E. Gutierrez-Ballesteros and D. H. Wenrich [181] considered it to be a free-living amoeba. In 1917, A. M. da Cunha and O. da Fonseca [183] described *E. serpentis* from snakes (false jararacá, *Palusophis bifossatus* [=*Drimobius bifossatus*]) in Brazil, expanding the description the following year [222]. According to the authors, this species was morphologically similar to *E. testudinis* (a species originally described in turtles, see below) but differed mainly in its smaller size and host species. This proposal was questioned by Q. M. Geiman and H. L. Ratcliffe [184], who regarded *E. serpentis* as a synonym of *E. testudinis*. However, three decades later, in 1968, after examining the original material used by da Cunha and da Fonseca, T. N. Ghosh [223] left the question unresolved, pending re-examination of the original host species from which *E. serpentis* had been described.

In lizards (*Varanus*, *Tiliqua*) and snakes (*Pseudoboa*, *Lampropeltis*, *Natrix*) kept at the Philadelphia Zoological Garden (USA), Ratcliffe and Geiman [224] detected pathogenic amoebae with cysts described as “very similar to those of *E. histolytica*”, although they did not assign them to a specific species. In the same year, J. Rodhain [182] described *E. invadens* from snakes housed at the Antwerp Zoo (Belgium). This species is morphologically indistinguishable from *E. histolytica*/*E. dispar* but does not grow in culture at temperatures above 33 °C. While pathogenic in snakes and lizards, *E. invadens* appears to behave as a commensal in turtles, causing no apparent pathology [225].

Because of its relevance to the maintenance of reptiles in zoological collections, laboratories, and breeding facilities, *E. invadens* has been the subject of numerous studies, including experimental infections, culture-based work, and biochemical analyses. Its small-subunit ribosomal RNA (SSU rRNA) gene sequence is available [133,134]. Some DNA-based studies have suggested the existence of two genotypes or possibly two species within *E. invadens* [226], although this hypothesis has not been thoroughly investigated. Genetic analyses have also identified *E. invadens* in Komodo dragons [202]. More recently, a case was reported of a ball python that died after a short period of weight loss; necropsy revealed necrotizing colitis with intralesional *Entamoeba* trophozoites, which were identified by molecular analysis as *E. ranarum* [227].

*Entamoeba cuautlae* was described from Mexican lizards by Hegner and R. Hewitt [179]. These authors limited themselves to describing the new species without carrying out any comparative analysis with other compatible species recognized at that time.

In turtles, the first species described was *E. testudinis*, reported from Greek tortoises (*Testudo graeca*) imported into Brazil (the original geographic origin was not specified) in 1910 [189]. In the original work, only trophozoites were described. However, in 1912 A. Alexeieff found amoebae in Indian black turtles (*Melanochelys* [=*Nicoria*] *trijuga*) in Ceylon, identified them as *E. testudinis*, and described the cysts as tetranucleate [228]. Nevertheless, based on the figures provided in Alexeieff’s publication, Geiman and Ratcliffe [184] estimated the size range of the trophozoites in that study and noted that they were much smaller than those in Hartmann’s original description of *E. testudinis*. Consequently, both Alexeieff’s specific identification and the inclusion of *E. testudinis* within the histolytica group are questionable. It has been suggested [229] that the amoebae described by Alexeieff may in fact correspond to *E. barreti*, another species from turtles characterized by smaller trophozoites than *E. testudinis*, but for which cyst morphology has not been described [230].

Also in turtles, E. P. Sanders and L. R. Cleveland described another species, *E. terrapinae*, from specimens of the genus *Chrysemys* [187]. In the same host, they also identified (and temporarily cultured) trophozoites of *E. testudinis*, although encystation was not achieved. The distinction between *E. terrapinae* and *E. testudinis* is clear when trophozoite size is compared, being 10–15 µm in length in *E. terrapinae* and 50–70 µm in *E. testudinis*.

The most recently described turtle species assigned to the histolytica group were *E. insolita*, *E. testudinea*, *E. jaboti* and *E. knowlesi*. In the case of *E. insolita*, a clear diagnostic character is the structure of the cyst, which bears a thick, irregular, gelatinous-looking wall [185]. In 1944, A. Carini described two species from e Brazilian giant tortoise (*Chelonoidis denticulatus* [=*Testudo tabulata*]), *E. testudinea* and *E. jaboti* [188]. In both cases, the cysts (with only one or two nuclei in the case of *E. jaboti*) were described as having an external layer with irregular contours that appears to be somewhat viscous, as foreign bodies frequently adhere to it; this description is consistent with that of *E. insolita*. Despite this, J. Rodhain and M. T. van Hoof [186] considered both species different (they did not mention *E. jaboti*) when they described *E. knowlesi* from the eastern box turtle (*Terrapene carolina* [=*Terrapina cinosternoides*]) and the big-headed turtle (*Platysternon megacephalum*). *Entamoeba knowlesi* was differentiated from the other tetranucleate cyst-forming species infecting turtles mainly on the basis of trophozoite and cyst size.

In addition to *E. invadens*, SSU rRNA gene sequences are also available for *E. terrapinae* and *E. insolita*, as well as for several putative but unnamed taxa identified as ribosomal lineages (RL) or conditional lineages (CL) (see Section 8), including *Entamoeba* sp. from *Iguana iguana* [134] (identified as RL6 [173]), *Entamoeba* sp. RL5 from the leopard tortoise [173], and *Entamoeba* sp. CL1 from the giant Aldabran tortoise [202]. In addition, in a study analysing SSU rRNA sequences obtained from isolates from several turtle species kept in a herpetarium at the National Autonomous University of Mexico [231], animals were found to be infected with *E. terrapinae*, *E. invadens*, and *E. moshkovskii* in single or mixed infections. Within *E. terrapinae*, two distinct subgroups were identified, one corresponding to this species and the other designated *Entamoeba* sp. CL2 [202].

Among the *Entamoeba* species reported from fish, three form tetranucleate cysts: *E. salpae* (originally described as *Proctamoeba salpae*) in salema porgy (*Sarpa* [=*Box*] *salpa*) [38], *E. ctenopharyngodoni* [194], described from freshwater cyprinids in China, and *E. nezumia* [195], described from a deep-sea fish, *Nezumia bairdi*, collected off the coast of Greenland. There has been some controversy about the number of nuclei in the cysts of *E. salpae*. Alexeieff described the species as having cysts containing 4 nuclei (“J’ai observé les kystes à 4 noyaux (et à corps chromatoides)” [38] (p. 66)). However, E. R. Noble y G. A. Noble [232] considered its cysts had 4 to 8 nuclei, the mistake most probably derived from the definition of the genus *Proctamoeba* gave by Alexeieff, as “Kystes à 4 ou 8 noyaux” [38] (p. 71). As *Proctamoeba* was proposed in substitution of *Entamoeba* during the *Endamoeba-Entamoeba* controversy, the definition was intended to include both tetranucleate and octonucleate cyst forming species (i.e., *E. histolytica* and *E. coli*). Willbur J. Bullock [233] considered it to be a species forming only tetranucleate cysts, with occasional supernumerary nuclei (a phenomenon also reported sporadically in *E. coli* [14] and *E. polecki* [234]). However, again J. D. Orias and E. R. Noble [195] regarded octonucleate cysts as the normal form in *E. salpae*.

*Entamoeba ctenoparhingodoni* was characterized by the presence of cysts, a differential character with respect to the other species described at that time from freshwater fish (*E. pimelodi*, ref. [235]). In the case of *E. nezumia*, the marine habitat of the host was used as justification to consider this species distinct from those described from freshwater fish; the trophozoites were smaller than those of *E. salpae*, and the number of nuclei in the cyst further differentiates it from other *Entamoeba* species described from marine fishes (see Section 7.2 and Section 7.3).

Finally, several species assigned to this group are considered free-living. In addition to *E. moshkovskii*, originally discovered in wastewater and, as discussed above, capable of infecting a broad range of hosts, *E. ecuadoriensis* [133] and *E. marina* [89] have also been described. In both cases, a free-living lifestyle together with clear genetic differences from other species of the genus have been considered sufficient to justify their recognition as distinct species.

### 7.2. Entamoeba Species Forming Mature Octanucleate Cysts (Coli Group)

As in the case of species belonging to the histolytica group, abundant information is available for the human species, *E. coli*, whereas data for species described from other hosts are much more limited (Table 4). As discussed above, at the end of the 19th century and the beginning of the 20th century, Schaudinn [20] established the existence of two human intestinal species, *E. histolytica* (pathogenic) and *E. coli* (commensal), the latter characterized by the formation of octonucleate cysts. As occurred with *E. histolytica*, the taxonomic confusion of that period led to the description of several human species that were later regarded as synonyms of *E. coli* (e.g., *E. loeschi*, *E. williamsi*, *E. brasiliensis*) (Table 2).

Since Dobell’s monograph of 1919 [14], and with the exception of the erroneous proposal of *Councilmania*, all findings of octonucleate cyst-forming amoebae in humans and primates have been identified as *E. coli*. More recent studies have shown that genetic variability within this species is high and that cryptic species may be present; however, because the only differences detected so far are genetic, the prevailing approach has been to recognize the variants as subtypes within *E. coli* [201] (see Section 8). In Phayre’s leaf monkey (*Trachypithecus phayrei*), an isolate that would traditionally have been identified as *E. coli* was shown by genetic analysis to be more closely related to *E. muris* [173]. This lineage, designated *Entamoeba* RL7, has also been detected in humans [202].

Closely related to human and primate isolates of *E. coli* is *E. muris*, described in 1879 [21]. This species has been reported from a wide range of rodents and is morphologically indistinguishable from *E. coli*. It was accepted as a distinct species primarily on the basis of host association [14]. Sequencing of the SSU rRNA gene from isolates obtained from Mongolian gerbils has confirmed that *E. muris* is genetically distinct from *E. coli* [171]. As in the case of *E. coli*, it is possible that *E. muris* represents a complex of cryptic species. Shortly after the erection of *Councilmania* [5], Kessel [59,60] renamed *E. muris* as *Councilmania muris*, reassigned *E. muris decumani* from rats [246] as *Councilmania decumani*, and erected a new rodent species, *E. ratti*, based on supposed morphological differences between *Entamoeba* and *Councilmania* [60]. All these taxa were proposed as distinguishable on morphological grounds. These proposals were subsequently rejected after the genus *Councilmania* was considered invalid.

Another six *Entamoeba* species have been described from rodents, although one of them (*E. bobaci*) is considered separately in Section 7.5, as its cyst stage has not been described. Ernest L. Walker described several amoebae [240], including *Amoeba cobayae* from guinea pigs; however, based on the figures provided, it is likely that he described a mixture of organisms, and his proposals were subsequently regarded as invalid. Nevertheless, Chatton [247] accepted Walker’s description of the trophozoites and renamed the species *E. cobayae*. In the following year, Chatton [238] described *E. caviae* from guinea pigs. Dobell [14] regarded *E. caviae* as a junior synonym of *E. cobayae*. In contrast, Levine [73] rejected Walker’s original description and Chatton’s renaming and treated *E. caviae* as the valid species name. Dashu Nie, in his study of the intestinal protozoa of the guinea pig [248], consistently used the name *E. cobayae*, however in a single passage of the text, most likely due to a lapsus, the organism is referred to as *E. caviae*. *Entamoeba cobayae* was considered distinct from *E. muris* on the basis of chromatoid body morphology and negative results in experimental cross-infection studies [249], and was later identified primarily on the basis of host species [248]. Hoare [172], however, prioritized morphology over host association and synonymized *E. cobayae* with *E. muris*. Interestingly, despite the close morphological similarity between *E. muris* and *E. coli*, Hoare retained *E. muris* as a valid species, implicitly giving greater weight to host species as a differential criterion in this case.

*Entamoeba citelli* was described from squirrels (the thirteen-lined ground squirrel, *Ictidomys* [=*Citellus*] *tridecemlineatus*) by E. R. Becker [239], who differentiated it from *E. muris* on the basis of cyst wall thickness, a yellowish coloration of the cysts, and nuclear features, including dispersed chromatin. *Entamoeba dipodomysi* was described from kangaroo rats (*Dipodomys spectabilis*) by Hegner [242]; in the original description, only trophozoites were observed, and the justification for the new species was based solely on the host species. Several years later, D. J. Doran [250] described the cyst stage from infections in a congeneric host (*Dipodomys panamintinus*).

*Entamoeba marmotae* was described from the woodchuck (*Marmota monax*) by H. B. Crouch [243] and was considered similar to *E. citelli*, although differences in both trophozoite and cyst morphology were deemed sufficient to justify the erection of a new species. Subsequent findings in yellow-bellied marmots (*Marmota flaviventer*) were identified as *E. marmotae* on the basis of host species and trophozoite morphology, although cysts were not observed [251]. Finally, *E. criceti* was described from hamsters by Starkoff [241], with a morphology similar to that of *E. muris*.

The range of variability reported for *E. muris* was considered to encompass that of the other rodent species, which were therefore treated as synonyms of *E. muris* [237]. However, negative results obtained in experimental cross-infections led J. R. Hampton and A. W. Grundmann [252] to recognize *E. citelli*, *E. marmotae* and *E. dipodomysi* as distinct species. In general, Neal’s viewpoint [237] has prevailed, and rodent isolates have traditionally been identified as *E. muris* on the basis of morphological and host-associated criteria. Nevertheless, although the number of rodent isolates characterized by genetic analyses remains limited, available data indicate substantial genetic variability. In addition to the SSU rRNA gene sequence obtained from Mongolian gerbils, another sequence (*Entamoeba* sp. RL11) from a field vole (*Microtus agrestis*) has been reported [202], together with an unpublished sequence from a laboratory rat. These sequences are sufficiently divergent to suggest that *E. muris* may represent a species complex.

A further source of taxonomic uncertainty is the possible existence of lineages or species capable of infecting rodents as well as humans and NHPs. Supporting this possibility, Stensvold and colleagues [173] found an isolate from a chinchilla to be almost identical to one obtained from a gorilla. In addition, *Entamoeba* sp. RL7, detected in monkeys and humans, clusters phylogenetically with *E. muris*, and the sequence obtained from a laboratory rat isolate is identical to that of *E. coli* ST2 from humans. In the latter case, the initial identification of the rat isolate as *E. muris* was considered incorrect and reassigned to *E. coli* ST2, although no explicit justification beyond molecular identity was provided [202]. An alternative interpretation (that *E. coli* ST2 could correspond to a lineage primarily infecting rodents, potentially representing a new subtype of *E. muris*) was not explored. Given that morphological criteria do not allow clear discrimination between *E. coli* and *E. muris* [14,102], and that sequence identity is compatible with either interpretation, both possibilities remain plausible, and such assignments should therefore be regarded as tentative. The coexistence of cryptic species and partially overlapping host ranges may help to explain the results of experimental infections conducted by D. Owen [253], who found rodents to be susceptible to infection with human-derived *E. coli* in a single case; infections using 15 additional human and NHP isolates were negative.

In wild mammals, *E. bradypi* was described from a three-toed sloth (*Bradypus variegatus* [=*griseus*]) imported into the United States from Panama and dying three weeks later; the organism was observed post mortem and described as a new species on the basis of nuclear morphology [157]. Only a single cyst was observed in the original description, but additional data supporting the validity of the species were later provided from findings in two-toed sloths (*Choloepus didactylus*) [254].

In domestic mammals, *E. cuniculi* was described by Brug [42] from rabbits. No comparative analyses with other species were included in the original description, and given its morphological similarity to *E. coli* and *E. muris*, it was subsequently regarded as a synonym of *E. muris* by Neal [237] and Hoare [172]. *Entamoeba wenyoni* was described from goats and differentiated from other species known at the time by its small size (cysts 6–9 µm in diameter) [236].

Disregarding the descriptions by Walker [240], in which organisms other than amoebae may have been observed, only one species of the coli group has so far been described in birds, *E. gallinarum* in poultry, erected by E. E. Tyzzer in 1920 [244]. In Tyzzer’s original description, as well as in subsequent accounts by Hegner [214] and S. McDowell [255], the trophozoites of this species show no major differences from those of *E. coli*–*E. muris*, and the cyst size range overlaps with that of these species. The fact that the organism was found in an avian host was considered by Tyzzer sufficient justification to propose it as a distinct species. This interpretation appeared to be supported by the results of Hegner [214], who showed in experimental infections that *E. muris* could infect domestic chickens only transiently (2–3 days), whereas infections with material from turkeys and guinea fowl persisted over time.

More recently, F. Ponce-Gordo and colleagues [256] reported *E. coli*-like cysts in rheas bred in Spain. Although cyst size was larger than that reported by Tyzzer, McDowell and Hegner for *E. gallinarum*, and the host species was markedly different, no new species was proposed. A sequence is available from an octonucleate cyst-forming *Entamoeba* isolate obtained from lesser rheas (*Pterocnemia pennata*), but it has not been assigned to *E. gallinarum*. This sequence is identical to a partial sequence from a human isolate [201]. This is not the only example of *Entamoeba* species infecting both avian and mammalian hosts, as similar host-range overlap has been demonstrated by genetic analyses for some members of the bovis group (see Section 7.3).

No species of the coli group have been described from amphibians or fishes (see Section 7.1 for the discussion about *E. salpae* in fishes), but three have been described from reptiles: *E. lacerticoli* from Californian lizards [245], *E. flaviviridis* from Indian geckos [190] and Sudanese lizards [257], and *E. ctenosaurae* from iguanas (*Ctenosaura acanthura*) [179]. Wenyon [258] reported the presence of octonucleate cysts in sand lizards (*Lacerta agilis*) and starred agamas (*Laudakia* [=*Agama*] *stellio*) from Egypt, but did not assign them to a named species. The validity of these reptile species has neither been revised nor questioned.

### 7.3. Entamoeba Species Forming Mature Uninucleate Cysts (Bovis Group)

Most species assigned to this group were described during the first half of the 20th century, and the validity of many of them has been subject to debate (Table 5). Given their marked morphological similarity, host species has at times been treated as a decisive taxonomic criterion, depending on the authors. It is noteworthy that, whereas for species of the histolytica and coli groups infecting humans and NHPs the prevailing tendency was to regard isolates from different hosts as belonging to the same species (*E. histolytica* or *E. coli*), with genetic data later allowing the recognition of cryptic species or ribosomal lineages, the opposite trend prevailed for species of the bovis group. In this latter case, particularly among species infecting ungulates, the dominant approach during the same period was to propose distinct species according to host species or geographical origin, even when morphology (and sometimes host species) was essentially identical.

The first species of this morphological group was *Amoeba bovis* from cattle in Germany, described by E. Liebetanz in 1905 [261], based on trophozoites. Shortly thereafter, the species was redescribed and renamed *E. bovis* by Liebetanz himself [266] and by R. Braune [267], and its cysts were subsequently described by O. Nieschulz [268]. Swellengrebel [158] described *E. ovis* from sheep and *E. chattoni* from monkeys; both species were characterized as having nuclei with very similar features, with host species representing the main distinguishing criterion. Matiranjan Das Gupta, in 1935 [269], reported the presence of cysts 5–8 µm in diameter in the stomach of goats slaughtered at the Calcutta abattoir and identified them as *E. ovis*, although Hoare [270] later expressed doubts regarding this identification. Despite proposals that *E. ovis* might be a synonym of *E. bovis* [254], both species were retained as separate throughout the 20th century.

The first observation of an intestinal amoeba in pigs was reported by T. Smith [271] in animals from the United States. Smith initially regarded the organism as *E. coli*, but Hartmann re-evaluated the material and published the finding as *E. suis* [265]. One year prior to this proposal, von Prowazek [98] had described a uninucleate amoeba found in pigs and in a child from the Mariana Islands, which he named *E. polecki*. Prowazek’s description was considered inadequate by several authors, partly because more than one species may have been present in the material examined and because the description itself was ambiguous. Following the same reasoning applied to Walker’s *Amoeba cobayae*, Dobell [14], O. Nieschulz [263,272] and Hoare [270] proposed that the name *E. polecki* should be rejected and that *E. suis* should instead be accepted as the valid name for the species infecting pigs. However, the prevailing view was that the name *E. polecki* should be retained as valid [3,102,254,273,274]. Although Levine attempted to maintain the name *E. suis* over *E. polecki* [73,74], the majority of studies published since the mid-20th century have identified the species from pigs as *E. polecki*.

In German goats, Nieschulz [275] found amoebae that he considered to be the same species he had previously reported from pigs [272], and which he later proposed as a new species, *E. debliecki* [263]. To the debate on the validity of *E. polecki* vs. *E. suis*, which was largely based on whether or not von Prowazek’s description was accepted, *E. debliecki* was added as a further complicating factor, as it had also been described from pigs. With the exception of Wenyon [102] and Hoare [270], most authors involved in this debate regarded *E. debliecki* as a synonym of *E.polecki* [3,73,74,102,254,273,274]. More recently, some authors have analysed samples from English goats that were thought to correspond to *E. debliecki* [202].

It was already accepted that the *Entamoeba* species infecting goats was the same as that infecting pigs (*E. polecki*) when G. A. Noble proposed a new species, *E. antilocapra*, from pronghorn antelope (*Antilocapra americana*) kept in a zoo in the United States [259], and another new species from Philippine goats, *E. dilimani* [264]. In both cases, several morphological differences were reported, particularly in cyst size and nuclear morphology. The figures presented in both original descriptions show marked similarities to those of the invalidated *E. debliecki* illustrated by Hoare [270], as well as to those from goats identified by G. A. Noble and E. R. Noble [3] as *E. polecki*. Nevertheless, in the case of *E. dilimani*, the size differences were substantial and the species was considered to possibly have an Asian distribution distinct from that of the species infecting goats in Western countries; consequently, it was accepted as valid [73,74]. By contrast, the name *E. antilocapra* has not been used subsequently; later findings in pronghorn antelope were identified as *E. bovis*, and *E. antilocapra* was regarded as a synonym [260].

The most recently described species from bovids is *E. bubalus*, reported from water buffalo (*Bubalus bubalis*) in the Philippines in 1955 [262]. The differential characters used were nucleus morphology and host specificity, as no similar cysts were observed in other domestic animal species. This proposal was based on the examination of only two trophozoites and 23 cysts obtained from 12 animals (1–2 cysts per host), and the description closely matched that of *E. bovis* from cattle in the United States, Korea and Japan [3]. Nevertheless, the species was cited as valid by Levine in subsequent classical books [73,74,276].

In primates, Chatton [173] found intestinal amoebae in *Macacus sinicus*, and two years later Swellengrebel [158] described a new species, *E. chattoni*, from *Macacus rhesus*. This species was subsequently located, identified and confirmed in 14 primate species kept in zoos [159]. *Entamoeba chattoni* is morphologically almost indistinguishable from *E. polecki* [3,277], but it has been retained as a separate species owing to its different host range (NHPs vs. pigs, goats and humans for *E. polecki*). Biochemical [278] and genetic differences [133,134] have been described that allow *E. polecki* and *E. chattoni* to be distinguished and support their recognition as separate species.

By the end of the 20th century, under morphology- and host-based taxonomy, *E. bovis*, *E. ovis*, *E. dilimani*, *E. bubalus* and *E. polecki* were generally accepted as valid species in ungulates, and *E. polecki* and *E. chattoni* in humans and NHPs. Subsequent genetic analyses based on SSU rRNA gene sequences, performed over the last 25 years in a wide range of domestic and wild hosts, have so far failed to provide a clear resolution of the situation in ungulates and synonymies have been proposed.

The first sequences from species of this group (*E. polecki* and *E. chattoni*) were obtained in 1999 [134]. In a study published in 2001, J. J. Verweij and colleagues [279] identified four distinct SSU rRNA gene sequences in human isolates containing uninucleate *Entamoeba* cysts: one variant corresponded to *E. polecki*, another to *E. chattoni*, and the remaining two to unidentified species. One of these was later named *E. struthionis* [77]; originally described from ostriches, it has also been shown to infect humans and pigs [196,202,280,281,282]. Because of the close morphological similarity and shared host range among *E. polecki*, *E. chattoni* and *E. struthionis*, these taxa were proposed as synonyms [196]. Although this proposal was subsequently challenged [199], the species were later “downgraded” to ribosomal lineages within *E. polecki* [173,202]. More recently, they have been reinstated as distinct, valid species [283].

The species *E. suis* has also been resurrected in 2006, when a novel ribosomal sequence was found in Vietnamese pig isolates [196]. It has also been detected in pigs in China [284], Japan [285] and Germany [286], in gorillas in UK [173], and in zoo NHPs and ostriches in China [287].

The first published ribosomal sequences from *Entamoeba* species infecting bovids were obtained in 2010 and assigned to *E. bovis* [201]. These sequences were obtained from different hosts (sheep, cattle, reindeer and roe deer) and showed marked differences among isolates, as well as, in some cases, the presence of distinct sequences within the same sample. This inter- and intra-isolate variability has been consistently observed in most subsequent studies conducted on bovid samples, the majority of them carried out in China [51,203,204,205,206,207,208,209,211]. No specific sequence type has been found to be associated with a particular host species, leading to the proposal in 2016 that *E. ovis* should be regarded as a synonym of *E. bovis* [202]. *Entamoeba dilimani* and *E. bubalus* have not been explicitly discussed in this context, but by extension they could also be interpreted as synonyms. Although some of the detected variants (if not all) may correspond to distinct species [173,202], no attempt has yet been made to relate the genetic variants identified to the species historically described from bovids, possibly due to the lack of a clearly defined species concept and difficulties in their correct taxonomic assignment (see Section 8).

In birds, a species of this group, *E. struthionis*, has been described from ostriches [76,77], but infections of these birds by *E. suis* have also been reported [287]. In the common rhea (*Rhea americana*), both *E. polecki* and *E. struthionis* have been identified [173]. As noted above, *E. struthionis* was regarded as a synonym of *E. polecki* [196] or as a subtype of *E. polecki* [173] until it was recently reinstated as a valid species [283].

Three species of the bovis group have been described from poikilothermic animals. In amphibians, G. V. Epstein described *E. ilowaiskii* from frogs (probably *Rana temporaria*) in Russia [219]. In marine fish, *E. gadi* was described from the rectum of pollock or coalfish (*Pollachius virens*) captured in the English Channel [233], and *E. chiangraiensis* was described from the freshwater Asian swamp eel (*Monopterus albus*) in Thailand [90]. In these cases, host characteristics and ecosystem type, and in the case of *E. chiangraiensis* its SSU rRNA gene sequence, were the main criteria used by the respective authors to justify their taxonomic proposals.

### 7.4. Entamoeba Species Not Forming Cysts (Gingivalis Group)

Demonstrating that something exists is relatively straightforward, whereas demonstrating that something does not exist is inherently more difficult. The absence of a cyst stage in *E. gingivalis* has not been established through a single experimental demonstration but rather through more than a century of convergent morphological, ecological and experimental evidence. Since its original description (as *Amoeba gingivalis*) from dental calculus [6], *E. gingivalis* has been repeatedly and exclusively observed as a trophozoite in the human oral cavity, particularly in dental plaque and gingival pockets [288]. Despite extensive microscopic examinations of oral material from both healthy individuals and patients with periodontal disease, no structures consistent with true cysts (i.e., spherical stages with a defined cyst wall and a fixed, species-specific number of nuclei) have ever been documented.

As occurred with intestinal amoebae, during the second half of the 19th century numerous species of orally located amoebae were described in humans, based on minor morphological differences, but all of them were eventually regarded as synonyms of *E. gingivalis* [57,289]. A separate mention should be made of *E. pyogenes* described by Verdun and Bruyant in 1907. Although the original publication was not examined firsthand for this study, the description provided by A. J. Smith and M. T. Barrett in 1915 [289] based on the original work is striking in that it reports the presence of two types of cysts: tetranucleate cysts 6–15 µm in diameter and large uninucleate cysts (without size indication). It is likely that the structures identified as cysts by Verdun and Bruyant were of a different origin, and in Dobell’s opinion [14], *E. pyogenes* represents a synonym of *E. gingivalis*.

Although some authors went so far as to consider *E. gingivalis* a synonym of *E. histolytica* [289,290], the species has been largely accepted as distinct and this status has been confirmed by genetic analyses [133,134,291]. In 2018, a different genetic variant was detected in patients with and without periodontal disease in Mexico and was designated *E. gingivalis* ST2 (kamaktli strain) [292,293]. Both variants can occur simultaneously in the same patient [293]. More recently, a novel ST3 subtype, detected only in association with the ST1 subtype, has been identified in Austrian patients [294].

The only available data on *E. gingivalis* in NHPs derive from studies carried out during the first quarter of the 20th century. The species has been reported from the oral cavity of chimpanzees [295] and of captive and wild monkeys (rhesus monkeys and baboons) [296,297,298], and it was considered to be the same species following successful bidirectional cross-transmission experiments between monkeys and humans [297].

Whereas oral amoebae detected in NHPs have generally been identified as *E. gingivalis*, findings in other hosts have occasionally been proposed as distinct species, including *E. canibuccalis* [299], *E. equibuccalis* [300] and *E. suigingivalis* [301] (Table 6). Of these, *E. canibuccalis* was regarded as a synonym by Levine [73], given the morphological identity of the trophozoites, the successful experimental infection of dogs with human-derived amoebae [296], and the possibility of natural transmission between dogs and humans through close contact [73]. The other two species have been retained as valid, as they exhibit smaller trophozoites than *E. gingivalis* and potential transmission between different host species (human-animals) is not readily explained.

### 7.5. Species for Which the Cyst Stage Is Unknown

This group corresponds to the taxa designated by Levine as insufficiently known species. Species included in this category have generally been described on the basis of trophozoite morphology alone (Table 7). In some cases, authors have tentatively regarded these insufficiently known species as non-encysting ones (gingivalis group). However, it should be noted that the authors who originally described these species may simply have failed to detect or recognize a cyst stage that in fact exists; therefore, the absence of a cyst description does not necessarily imply the absence of a cyst stage.

We have included *E. barreti* in this group, although it is possible that this species may in fact be non-encysting. It was described by W. H. Taliaferro and F. O. Holmes [230] from turtles, based on fresh material and cultures maintained by Barrett and Smith [307]. The description relied exclusively on trophozoite morphology, as cysts were never observed. In a later isolate (from 1971), cysts were again not observed in vivo, nor could encystation be induced in vitro, even when cultures were subjected to procedures that successfully induced encystation in other reptile species [308]. For this reason, *E. barreti* could potentially be regarded as a non-cyst-forming species and assigned to the gingivalis group. Neal, however, placed it within the coli group on the basis of its locomotion pattern, characterized by little differentiation between ectoplasm and endoplasm [4]. In any case, *E. barreti* shows differential characteristics compared with other *Entamoeba* species described from reptiles (*E. testudinis*, *E. serpentis*, *E. terrapinae*, *E. invadens* and *E. insolita*). Among turtle-infecting species, trophozoites of *E. testudinis* are much larger, whereas *E. terrapinae*, *E. invadens* and *E. insolita* all form detectable cysts in faeces.

In the case of *E. caprae*, Fantham [303] observed only trophozoites, whose characteristics were compatible with those of *E. coli*, except for their larger size. Glenn A. Noble and E. R. Noble [3] considered that size alone was insufficient to justify the erection of a new species and therefore regarded *E. caprae* as invalid. Notably, G. A. Noble described other species on the basis of minor morphological differences (see Section 7.3).

In horses, L. Gedoelst [305] described a species as *Amoeba intestinalis*, which he reported from a wide range of animals (horses, pigs, cats and turkeys). In 1920 and 1921, Fantham described an amoeba from the intestine of horses and identified it as *E. intestinalis* Gedoelst [175,309]. Only trophozoites were observed, with characteristics consistent with *E. coli*. This species was later renamed *E. gedoelsti* [197], as the name proposed by Gedoelst was preoccupied by *Amoeba intestinalis* Blanchard, 1885, one of the many synonyms of *E. coli*. The validity of this species remains unresolved. In more recent studies, *Entamoeba* trophozoites (identified as *E. equi*) have been detected in equids, including horses [195,202] and zebras [201]. It has been suggested that the apparent absence of cysts in these samples may be related to their scarcity or to periodic shedding [202].

Li Yuan-Po described *E. bobaci* from marmots (*Marmota bobak*) [302]; no cysts were reported, and the proposal of a new species was based on comparisons with *E. coli*. Neal [237] regarded this species as a synonym of *E. muris*.

Three additional species have been described for which cysts have not been observed. In reptiles, *E. varani* was described from the Nile monitor (*Varanus niloticus*) [306]; the most similar species at the time was *E. ranarum*, which was rejected as an identification solely on the basis of host species. In fish, *E. pimelodi* was described from Brazilian freshwater fish (catfisch, *Pimelodus blochi* [=*clarias*]) [235] and *E. molae* from a marine fish (ocean sunfish, *Mola mola*) [232]. In both cases, no cultures were established and no attempts were made to induce encystation, leaving open the question of whether these amoebae are truly non-cyst-forming species or whether cysts simply went undetected.

### 7.6. Species for Which the Original Description Could Not Be Consulted

*Entamoeba elephantis* was described in a conference communication by D. Mandal and A. Choudhury in 1984 [304]. Only the abstract of this contribution could be accessed, and it provides no descriptive details; moreover, the species has not been cited in subsequent literature. More recently, *E. moshkovskii* has been identified in an African elephant in Namibia [310], and a novel ribosomal lineage (RL10), phylogenetically related to *E. hartmanni*, has been proposed based on an isolate obtained from an Asian elephant kept in a zoo in the Netherlands [202].

An unidentified *Entamoeba* sp. has been reported in whales [311], though no further data were provided. The number of nuclei cannot be definitively determined from the published images; while one nucleus is clearly evident, others are only vaguely discernible and appear out of focus.

## 8. Interpreting Species Delimitation in *Entamoeba*: Lessons from the Past and Consequences for Current Taxonomy and Diversity

Describing, delimiting, and naming species constitute what is traditionally referred to as alpha taxonomy. These processes are conceptually distinct and, as shown in previous sections, have not always been clearly separated in the taxonomic history of *Entamoeba*. Specimen identification and species discovery are conceptually different operations, as are species description and species delimitation [312]. Specimen identification refers to the assignment of an individual organism, or an isolate in the case of protists, to a previously described and named species based on existing diagnostic criteria and does not in itself test species boundaries but rather applies them [313]. Species discovery, by contrast, involves the recognition that a set of organisms cannot be satisfactorily accommodated within any known species and therefore represents an unrecognized taxon; in asexual organisms such as *Entamoeba*, this step is necessarily decoupled from reproductive criteria and instead relies on the detection of coherent, non-random biological variation consistent with independent evolutionary trajectories [314]. (It is generally accepted that species of *Entamoeba* reproduce exclusively asexually; although the possibility of sexual reproduction has been suggested [315,316], this hypothesis remains unconfirmed). Species delimitation entails the explicit formulation and evaluation of boundaries between newly identified lineages and established species, with the aim of determining whether the observed diversity reflects intraspecific variation or the existence of independently evolving entities. Under a lineage-based view of species, which is especially applicable to asexual organisms, delimitation is best understood as a hypothesis-testing process rather than the application of a single defining property [317]. Finally, species description constitutes the formal taxonomic act that follows delimitation, whereby a species hypothesis is made explicit through a diagnostic description, the designation of type material or reference entities, and the assignment of a valid scientific name in accordance with the relevant nomenclatural code. This step enables effective communication, comparability, and the accumulation of knowledge across studies [318,319].

The key issues are the species concept adopted for delimitation and the criteria used both to differentiate species and to identify specimens. During the 19th century and the first half of the 20th century, the taxonomy of *Entamoeba* largely followed the classical framework applied by taxonomists to most organismal groups. In practice, the decision as to whether two populations belonged to the same or to different species relied overwhelmingly on a morphological–typological species concept. Species were regarded as fixed, or at least relatively stable, “types” defined, in *Entamoeba*, by a few diagnostic characters, and taxonomic judgments were based on the recognition of apparent morphological or biological differences among forms.

This typological approach dominated descriptive taxonomy in general, and particularly protist taxonomy, where modes of reproduction were poorly understood or inaccessible to direct observation. As a consequence, species boundaries were inferred almost exclusively from observable traits and overall similarity, rather than from population-level processes or evolutionary relationships. In *Entamoeba*, the scarcity of reliable and consistent morphological characters led taxonomists to complement morphology with additional criteria, including physiological traits (host species, ability to grow in vitro, results of experimental cross-infections) and biogeographical information. These supplementary criteria, however, were not applied consistently and were often the subject of debate. A striking asymmetry emerges when comparing species associated with humans and NHPs with those infecting other vertebrates. In the former case, the absence of clear morphological differences promoted a conservative taxonomic approach, whereby multiple biologically distinct entities were subsumed under a single species name. In contrast, for species infecting ungulates, reptiles, fish and other hosts, minor morphological variation, host specificity, or geographic origin alone often sufficed to justify the description of new species, frequently in the absence of comparative analyses or information on the life cycle.

Historically, three main approaches can be identified for delimiting and accepting or rejecting species within this genus:

1. Use of a “complete set” of traits. Under this approach, isolates were considered to represent a distinct species if they differed from related taxa in one or more of the following characters: trophozoite or cyst morphology, host species, biological traits (pathogenicity, in vitro cultivation, cross-infection experiments), biochemical or genetic markers, or geographic origin (see Section 2, Section 6 and Section 7). Host species, sometimes combined with geographic origin, was frequently used as a decisive criterion. Notably, even the same authors applied this criterion inconsistently (e.g., G. A. Noble synonymized *E. debliecki* from Western goats while almost simultaneously erecting *E. dilimani* in Eastern goats, *E. antilocapra* in antelope, and *E. bubalus* in water buffalo; see Section 7.3).
2. Use of a “partial set” of traits (option 1). In this framework, morphological, biological and biochemical differences were considered valid for species delimitation, whereas host species and geographic origin were regarded as insufficient on their own. These latter criteria could support a species description but were not accepted as primary delimiters.
3. Use of a “partial set” of traits (option 2). This approach is similar to the previous one but applies a stricter threshold: only clear and substantial biological or biochemical differences are considered taxonomically meaningful, whereas minor differences are dismissed. This option necessarily introduces a degree of subjectivity in defining what constitutes a “small” or “insufficient” difference. It was applied, for example, to propose *E. chattoni* and *E. struthionis* as synonyms of *E. polecki* (see Section 7.3).

A recent proposal has argued that lineage-based species concepts, such as the general or unified species concept [317] and the pragmatic species concept [320], are particularly appropriate for *Entamoeba* [283]. Under this framework, a species of *Entamoeba* is defined as an independently evolving metapopulation lineage, delimited through the integration of morphological, ecological, genetic, and host-related evidence, in accordance with the principles of integrative taxonomy [321]. In practice, the criteria currently applied in *Entamoeba* taxonomy [173] are largely compatible with this conceptual framework, although some modifications and refinements are required (see below).

Morphological criteria have never been fundamentally questioned and remain central to species identification. However, within each of the traditional morphological groups (bovis, histolytica, coli, gingivalis), interspecific morphological differences are often minimal or absent, making cryptic diversity a recurrent issue. By contrast, the remaining criteria historically used for species delimitation have been controversial to varying degrees.

The geographic origin of an isolate was occasionally invoked by some authors as a differentiating character (for example, for some species of the bovis group, see Section 7.3), but it has ultimately not been accepted as a valid criterion for species delimitation, at least in humans and domestic or human-associated animals. Human migration, animal trade, and the widespread translocation of livestock and companion animals have likely facilitated the global dissemination of their associated *Entamoeba* species, thereby eroding any meaningful biogeographic signal at the species level.

Most controversial is the use of the host criterion. As noted in the previous sections, some authors have considered the host–parasite relationship as rigid while others considered morphologically similar parasites in different host species as belonging to a single species. The best option is that pointed out by J. F. Kessel [322] that each host–parasite species relationship should be considered individually. To avoid an uncontrolled proliferation of species based solely on host association, authoritative taxonomic revisions by leading specialists adopted a restricted use of host species as a differential criterion [3,14,172,270]. Host identity was considered taxonomically informative when the hosts belonged to clearly distinct zoological groups (e.g., *E. gallinarum* in birds, *E. coli* in humans, and *E. muris* in rodents); in contrast, when hosts were closely related or belonged to the same broad zoological group, host species alone was not regarded as sufficient for species delimitation and had to be supported by consistent morphological differences, or the nominal species were treated as synonyms (e.g., *E. coli* = *E. pitheci*; *E. polecki* = *E. debliecki*; *E. histolytica* = *E. dispar*).

The inconsistent application of this criterion by different authors (sometimes even within the work of a single author) has contributed substantially to recurring taxonomic problems in the genus. An additional complication is that several *Entamoeba* species are capable of infecting a broad range of hosts belonging to clearly divergent zoological groups (e.g., *E. moshkovskii* in both poikilothermic and homeothermic animals; see Section 6). Conversely, a single host species may harbour multiple *Entamoeba* species simultaneously; humans, for instance, can be infected by up to ten different species. As a consequence, species erected primarily on the basis of host specificity have frequently been questioned or rejected in later taxonomic revisions (see, for example, the discussion of rodent-associated species in Section 7.2). Conversely, the opposite practice (synonymizing taxa solely on the basis of a shared host range) has also generated controversy and disagreement [199].

Biochemical and genetic data have often been regarded as the solution to the problem of cryptic species. Indeed, the application of these approaches made it possible to separate and formally recognize as distinct species *E. dispar*, *E. bangladeshi* and *E. nuttalli* from *E. histolytica* (see Section 6), *E. ecuadoriensis* from *E. moshkovskii* (Section 7.1), and *E. suis* from *E. polecki* (Section 7.3). However, the widespread use of genetic analyses (primarily SSU rRNA gene sequences) in epidemiological and ecological studies has revealed an unexpectedly high level of genetic diversity within *Entamoeba*.

In response to this situation, recent authors have deliberately avoided proposing new species based solely on genetic divergence [133], particularly when the detected lineages share the same host range [196]. Instead, a non-standard subspecific nomenclature has been adopted to accommodate *Entamoeba* diversity without formal taxonomic inflation [173,202,210]. Under this framework, several categories have been introduced: subtypes (ST), defined as phylogenetic clusters within a formally recognized species [173]; ribosomal lineages (RL), corresponding to isolates forming distinct phylogenetic branches [173]; conditional lineages (CL), conceptually equivalent to RLs but based on partial SSU rRNA gene sequences obtained by Sanger sequencing [202]; and sequence types (SQT), analogous to CLs but inferred from next-generation sequencing data [210]. To date, eleven ribosomal lineages have been identified [202].

This system has proven useful as an operational tool to manage the rapidly growing volume of sequence data. Nevertheless, because RLs and CLs may ultimately correspond to biological species, the criteria used for their definition warrant re-evaluation. As originally conceived, these categories are defined primarily by their position as branches in phylogenetic trees, which in turn are determined by sequence similarity, largely independent of the specific tree reconstruction method employed [323]. Many taxonomists have criticized approaches such as DNA barcoding or algorithmic species-partitioning methods precisely because they rely on similarity thresholds rather than on diagnostic characters, whether qualitative or quantitative [312,321,324,325]. This same conceptual limitation applies to the current definitions of RLs and CLs.

To address this issue, a modification has recently been proposed [283] based on a qualitative diagnostic criterion: the presence of compensatory base changes (CBCs; both bases of a paired site in the SSU rRNA molecule mutate while the pairing itself is maintained [326]). This approach avoids reliance on arbitrary similarity thresholds and provides explicit diagnostic characters suitable for species delimitation. Its main limitations are that sequencing of the complete SSU rRNA gene may be required (the expansion region ES10 has been suggested [327], but no universally applicable barcode region can be defined in this gene in *Entamoeba* [283]), and that it may fail to discriminate very recently diverged lineages (e.g., *E. histolytica*–*E. nuttalli*), for which alternative loci may be more informative. Nevertheless, this framework eliminates subjectivity and meets the methodological requirements traditionally demanded by taxonomists.

This nonstandard nomenclature based on RLs and CLs should not be the definitive taxonomical option but a temporal solution while the data needed for species naming is obtained. The Code [69] requires a description of the species using characters stated in words (Art. 13.1.1) but nothing is said about its nature: it can be a morphological description (this is considered necessary by Stensvold and colleagues [173,202]) but also a gene description could be valid instead. In any case, it is mandatory the species name should be anchored to a name-bearing type (Arts. 72 and 73), which can be hapantotypes but also drawings, illustrations or micrographs which have been widely used and accepted in *Entamoeba* taxonomy.

There is an ongoing debate regarding the use of total DNA extracts as name-bearing types and the possibility of including them within the provisions of the different International Codes of Nomenclature [328]. In Zoology and Protistology, Art. 72.5.1 of the Code defines eligible name-bearing types broadly as “an animal [including microeukaryotes], or any part of an animal”, which could, in principle, allow a physically deposited total DNA extract (traceable to the sampled individual and curated in a recognized repository) to be interpreted as “part of an animal”. By contrast, DNA sequences themselves, which are obtained through copying processes such as PCR prior to sequencing, cannot serve as types but are valid as a description of the organism’s DNA [329]. However, the applicability of this provision to isolated DNA extracts remains debated, as this interpretation is not explicitly established under the Code and does not represent current standard practice. At present, modifications of the Code to allow DNA sequences to be used as name-bearing types are not under consideration [330].

Even if the Code were interpreted to allow a total DNA extract traceable to a sampled individual to serve as type material, this requirement is difficult to meet in *Entamoeba*, and the designation of new *Entamoeba* species based on DNA extracts as types could generate future taxonomic controversy. Many *Entamoeba* species cannot be cultivated in vitro, and DNA is typically obtained from faecal samples, which can be regarded, for the purposes of this discussion, as environmental samples. DNA extracted from such material (eDNA) represents a mixture of genetic material from multiple organisms, potentially including more than one *Entamoeba* species if cases of mixed infections. Under the current Code, both environmental samples and eDNA are therefore problematic as potential type material, because it may be difficult or even impossible to unambiguously associate the DNA with the organism of interest and to exclude co-occurring taxa [328].

In absence of the name-bearing type, the RL terminology seems to be for the moment the best alternative available. A subject of future debate will be the nomenclature of new species erected from RLs or other newly recognized lineages. In this review, we have listed a large number of *Entamoeba* species that, although validly named and published, were in some cases erected without adequate comparison with other species in the genus or lacked a name-bearing type, as no image or drawing was included in the original publication. In some instances, these species were subsequently redescribed, whereas in others no additional descriptions have ever been provided. In many cases, regardless of the quality of the original description, they were later treated as synonyms. Because synonymies are taxonomic hypotheses rather than nomenclatural acts, a name previously regarded as a subjective synonym may be reinstated if new evidence supports its recognition as a distinct species, in accordance with the principle of priority (the Code, Art. 23). Such reinstatement does not constitute a new description, and the authorship and date remain those of the original publication. However, redescriptions and, where necessary, the designation of a neotype (the Code, Art. 75) may be required to stabilize the application of the name. It should be emphasized that a redescription does not in itself imply the establishment of a neotype. Moreover, the type locality and, in the case of parasites, the host species are those of the original type, and any neotype should originate “as nearly as practicable from the original type locality and, where relevant, from … the same host species as the original name-bearing type” (the Code, Art. 75.3.6). In the study of Clark and colleagues [196], they resurrected the name *E. suis* for an *Entamoeba* species detected in pigs from Vietnam. In that work, the authors added a redescription based on morphological and genetic data and provided new images. While this approach supports the biological coherence and host association of the species, it does not satisfy the geographical requirements for neotype designation under the Code, because *E. suis* was described in pigs from the United States [271]; consequently, the Vietnamese isolate is best regarded as representative material supporting species recognition and the reinstatement of the name, but it cannot be considered a formal neotype. In the same study, the authors applied the name *E. equi* to an *Entamoeba* isolate from a horse. This species was originally described by Fantham [175], but no name-bearing type was designated: the original description did not include drawings or photographs, and if a hapantotype was ever available, it is no longer accessible. As no images of *E. equi* were provided by Clark and coworkers, the genetic data they presented contribute additional information but do not resolve the typification problem. Consequently, *E. equi* currently lacks a holotype or syntypes and should be regarded as a *species inquirenda*, that is, a species of doubtful identity requiring further investigation.

Another problem arises when newly detected lineages cannot be confidently assigned to any previously described species, whether synonymized or not. For example, several *Entamoeba* species were historically described from different ruminant hosts and later synonymized on morphological grounds (see Section 7.3). Genetic analyses now demonstrate that the morphospecies *E. bovis* comprises multiple cryptic lineages that are not host-specific and may occur across several ruminant species. In such systems, where host specificity is broad and original descriptions were based on limited or ambiguous diagnostic information, the application of historical species names to newly delimited cryptic lineages is not straightforward. On the one hand, the occurrence of a lineage in the same general host group from which a species was originally described may support the pragmatic reuse of an available name, particularly if there is no evidence that multiple lineages co-occurred in that host at the time of the original description. On the other hand, the absence of verifiable links to name-bearing type material, combined with the recognition of multi-host cryptic diversity, introduces unavoidable uncertainty. Under these conditions, the reuse of historical names represents a taxonomic hypothesis rather than a definitive assignment, and alternative approaches, including the introduction of new names, may be equally justifiable. We therefore consider that decisions regarding name reuse should be made on a case-by-case basis, with explicit acknowledgment of the underlying assumptions and of their potential implications for nomenclatural stability.

This historically oriented review shows that the taxonomy of *Entamoeba* has been shaped not only by the scarcity of robust diagnostic criteria and by differing opinions on their applicability but also by incomplete awareness of earlier literature. Several species names proposed in the late 19th and early 20th centuries subsequently fell into obscurity, occasionally leading later authors to assign newly detected isolates to a single available name simply because alternative names were unknown. In some cases, authors argued pragmatically for the continued use of an existing name in order to avoid leaving an organism unnamed (as in the case of *E. equi*; see Section 7.1). Although such decisions reflect a legitimate concern for terminological continuity, they also illustrate how the absence of a comprehensive historical framework can constrain taxonomic interpretation. By recovering and reassessing overlooked species names, the present review clarifies that more than one historical taxon may be applicable in some cases, thereby exposing the limitations of name assignment based primarily on convenience rather than explicit evidence. Even when these legacy names cannot be applied unambiguously, their recognition is essential to prevent the uncritical consolidation of distinct biological entities under a single name and to ensure that future taxonomic decisions are made within a complete, transparent, and historically informed nomenclatural context.

## 9. Toward an Operational Framework for Species Delimitation and Nomenclature in *Entamoeba*

Based on the historical analysis presented in this review and on current methodological possibilities, it is evident that future progress in the taxonomy of *Entamoeba* requires an explicit, coherent, and operational framework for species delimitation, identification, and naming. Such a framework must reconcile three constraints that have historically been difficult to integrate in this genus: (i) extreme morphological conservatism and cryptic diversity, (ii) frequent host sharing and incomplete host specificity, and (iii) a complex legacy of historical names of uneven diagnostic value. We propose the following general principles as a pragmatic synthesis to guide future taxonomic work on *Entamoeba*.

First, species delimitation should be explicitly framed within a lineage-based species concept, in which species are understood as independently evolving metapopulation lineages. Under this view, any line of evidence that supports evolutionary independence (such as molecular, morphological, ecological, or biological data) may be sufficient to support species delimitation. In *Entamoeba*, however, as shown in previous sections and it is indicated below, neither host species nor geographic origin should be regarded as valid criteria on their own for species delimitation, and morphology is frequently conserved across divergent lineages, such that genetic divergence often represents the only informative criterion. The use of multiple, congruent sources of evidence strengthens species hypotheses and remains desirable where feasible, but the absence of corroboration from additional data does not preclude species-level divergence based solely on genetic data in asexual protists. Rather, the decisive factor is whether the available evidence (genetic or otherwise) demonstrates evolutionary independence.

Second, morphological criteria should continue to be used as a primary tool for specimen identification and for assigning isolates to one of the traditionally recognized morphological groups (histolytica, coli, bovis, gingivalis). However, given the near absence of reliable interspecific morphological differences within these groups, morphology alone should not be considered sufficient for species delimitation except in rare cases where clear and consistent differences are demonstrable (for example, distinctive cyst structure, or clear size differences).

Third, host species and geographical origin should be regarded as contextual but not decisive evidence. Both criteria may contribute to species delimitation when supported by additional independent evidence, especially when infections are restricted to distinct zoological groups or to defined regions. Host specificity alone should not justify the erection of new species, nor should shared host range automatically lead to synonymization. The demonstrated capacity of several *Entamoeba* species to infect multiple, phylogenetically distant hosts, and the frequent occurrence of multiple species in a single host, preclude a simplistic host-based taxonomy.

Fourth, molecular data should play a central role in species delimitation, but their use must go beyond measures of overall sequence similarity. While SSU rRNA gene sequences have proven invaluable for revealing hidden diversity, the mere presence of divergent lineages is not, by itself, sufficient to define ribosomal lineages or species. Qualitative molecular criteria, such as the presence of compensatory base changes in conserved regions of the SSU rRNA secondary structure, provide a more robust, reproducible, and conceptually sound basis for delimiting independently evolving lineages. Qualitative criteria reduce subjectivity and align molecular evidence with diagnostic principles traditionally required in taxonomy.

Fifth, the current use of informal categories such as subtypes, ribosomal lineages, conditional lineages, and sequence types has been useful as an interim solution to manage increasing genetic data. However, because many of these entities likely correspond to biological species rather than intraspecific variants, their definitions and taxonomic status should be explicitly re-evaluated. Lineages supported by consistent qualitative molecular differences should be treated as candidate species and formally assessed under an integrative framework, rather than remaining indefinitely in a provisional nomenclatural limbo.

Finally, nomenclatural decisions must be grounded in both biological evidence and historical rigor. When a newly delimited lineage can be reasonably linked to a previously described species (based on host association, morphology, geography, and the absence of conflicting evidence) the reuse and reinstatement of an available name should be preferred, in accordance with the principle of priority. Such actions should be accompanied by detailed redescriptions and, where necessary, by the designation of neotypes following the provisions of the International Code of Zoological Nomenclature. Conversely, when historical names cannot be applied with confidence, the erection of new species names is justified and may be preferable to forced or ambiguous name reuse. In all cases, the underlying assumptions and uncertainties should be stated explicitly.

In conclusion, the long and often contentious taxonomic history of *Entamoeba* reflects not only methodological limitations but also shifting conceptual frameworks and incomplete engagement with earlier literature. By integrating historical analysis with modern molecular approaches and explicit species concepts, it is now possible to move toward a more stable, transparent, and biologically meaningful taxonomy. The framework outlined here does not aim to provide rigid rules but rather a coherent set of guiding principles to ensure that future taxonomic decisions in *Entamoeba* are reproducible, evidence-based, and consistent with both evolutionary theory and nomenclatural practice.

## Figures and Tables

**Table 1:** Intestinal and oral Endamoebidae species described between 1912 and 1954, during the *Entamoeba* vs. *Endamoeba* controversy. This list includes species that were later considered synonyms.

Described as *Endamoeba*	Described as *Entamoeba*	Described Under Other Genera
	*E. hartmanni* Prowazek 1912	*Proctamoeba salpae* Alexeieff 1912
	*E. pitheci* Prowazek 1912	
	*E. polecki* Prowazek 1912	
	*E. duboscqi* Mathis 1913	*Loschia duboscqi* Mathis 1913
	*E. suis* Hartmann 1913	*L. legeri* Mathis 1913
	*E. chattoni* Swellengrebel 1914	
	*E. ovis* Swellengrebel 1914	
	*E. cercopitheci* Macfie 1915	
*E. hominis* Pestana 1917	*E. cobayae* (*Amoeba cobayae* Walker 1908) Chatton 1917	
*E. intestinivulgaris* Aragao 1917	*E. minutissima* Brug 1917	
	*E. tenuis* Kuenen and Swellengrebel 1917	
	*E. caviae* Chatton 1918	
	*E. cuniculi* Brug 1918	
	*E. serpentis* da Cunha and da Fonseca 1918	
	*E. gallinrum* Tyzzer 1920	
	*E. paradysenteria* Chaterjee 1920	
	*E. equi* Fantham 1921	*Councilmania lafleuri* Kofoid and Swezy 1921
	*E. caprae* Fantham 1923	*C. decumani* Kessel 1923
	*E. multinucleata* Mello 1923	*Caudamoeba sinesis* Faust 1923
*E. barreti* Taliaferro and Holmes 1924	*E. anatis* Fantham 1924	*Karyamoebina falcata* Kofoid and Swezy 1924
*E. ratti* Kessel 1924	*E. ateles* (*Ameba ateles* Eichhorn and Gallagher 1916) Suldey 1924	
	*E. debliecki* Nieschulz 1924	
	*E. dispar* Brumpt 1925	
*E. dipodomysi* Hegner 1926	*E. citelli* Becker 1926	
	*E. pimelodi* da Cunha and Penido 1926	
*E. bradypi* Hegner and Schumaker 1928	*E. varani* Lavier 1928	*Councilmania dissimilis* Kofoid 1928
International Commission on Zoological Nomenclature, 1928: decision 99, *Endamoeba* (=*Entamoeba*)		
*E. gedoelstii* Hsiung 1930	*E. terrapinae* Sanders and Cleveland 1930	
	*E. invadens* Rodhain 1934	
	*E. lacerticoli* Wood 1935	
	*E. wenyoni* Galli-Valerio 1935	
*E. marmotae* Crouch 1936		
*E. insolita* Geiman and Wichterman 1937		
	*E. canibuccalis* Simitch 1938	
	*E. equibuccalis* Simitch 1938	
*E. ctenosaurae* Hegner and Hewitt 1940	*E. pyrrhogaster* Lobek 1940	
*E. cuautlae* Hegener and Hewitt 1940		
	*E. ilowaiskii* Epstein 1941	
	*E. moshkovskii* Tshalaia 1941	
	*E. jaboti* Carini 1944	
	*E. testudinea* Carini 1944	
	*E. knowlesi* Rodhain and Hoof 1947	
	*E. caudata* Carini and Reichenow 1949	
	*E. dilimani* Noble 1954	
International Commission on Zoological Nomenclature, 1954: decision 312, *Endamoeba* and *Entamoeba* as different genera		

**Table 3:** Four-nucleate cyst-forming *Entamoeba* species (histolytica group) described since 1903. Some species were originally described under the genera *Amoeba*, *Endamoeba*, *Proctamoeba* or *Loschia*. Species previously described by Grassi [21] and Gros [6] that are currently regarded as valid are also included. Species described from human samples that are currently considered synonyms of *E. histolytica* or *E. hartmanni* are not included. Species are grouped by host category and ordered alphabetically. NHPs—non-human primates.

Original Species Epithet	Type Host (Other Hosts)	Comments
*E. ateles* [152]	monkeys	Synonymized to *E. histolytica* [14]
*E. bangladeshi* [166]	humans	
*E. cercopitheci* [151]	monkeys	Synonymized to *E. histolytica* [14]
*E. cynomolgi* [154]	monkeys	Synonymized to *E. histolytica* [156]
*E. dispar* [108]	humans (NHPs)	
*E. duboscqi* [150]	monkeys	Synonymized to *E. histolytica* [14]
*E. hartmanni* [40]	humans (NHPs)	
*E. histolytica* [20]	humans (NHPs)	
*E. nuttalli* [149]	monkeys (humans)	Synonymized to *E. histolytica* [14], resurrected in 2007 [163]
*E. sinici* [174]	monkeys	Synonymized to *E. histolytica* [14]
*E. equi* [175]	horses (zebras)	
*E. caudata* [176]	dogs	Synonymized to *E. histolytica* [172]
*E. anatis* [177]	ducks	
*E. lagopodis* [178]	grouse	
*E. cuautlae* [179]	lizards	
*E. lacertae* [180]	lizards	Free-living amoeba [181]
*E. invadens* [182]	snakes (turtles, Komodo dragons)	
*E. serpentis* [183]	snakes	Synonymized to *E. testudinis* [184]
*E. insolita* [185]	turtles	
*E. knowlesi* [186]	turtles	
*E. terrapinae* [187]	turtles	
*E. testudínea* [188]	turtles	
*E. jaboti* [188] *	turtles	
*E. testudinis* [189]	turtles	Synonymized to *E. barreti* [190]
*E. ranae* [191]	frogs	Synonymized to *E. ranarum* [14]
*E. ranarum* [21]	frogs (snakes)	
*E. pyrroghaster* [192]	salamanders	Synonymized to *E. ranarum* [193]
*E. ctenopharyngodoni* [194]	freshwater cyprinids	
*E. nezumia* [195]	marine gadiform fishes	
*E. salpae* [38]	marine sparid fishes	
*E. ecuadoriensis* [133]	wastewater	
*E. marina* [89]	marine sediments	
*E. moshkovskii* [53]	wastewater (humans, NHPs, pigs, elephants, snakes; insects?)	

* This species was described as forming cysts with 1 or 2 nuclei. However, its morphological characteristics, specifically a gelatinous-appearing covering, coincide with those of *E. testudinea*, and both resemble those of *E. insolita*.

**Table 4:** Eight-nucleate cyst-forming *Entamoeba* species (coli group) described since 1903. Some species were originally described under the genera *Amoeba*, *Endamoeba* or *Loschia*. Species previously described by Grassi [21] that are currently regarded as valid are also included. Species described from human samples that are currently considered synonyms of *E. coli* are not included. Species are grouped by host category and ordered alphabetically. NHPs—non-human primates.

Original Species Epithet	Type Host (Other Hosts)	Comments
*E. coli* [21]	humans (NHPs)	
*E. legeri* [150]	monkeys	Synonymized to *E. coli* [169]
*E. multinucleata* [168]	orangutans	Synonymized to *E. coli* [169]
*E. pitheci* [40]	orangutans	Synonymized to *E. coli* [169]
*E. wenyoni* [236]	goats	
*E. cuniculi* [42]	rabbits	Synonymized to *E. muris* [237]
*E. caviae* [238]	guinea pigs	Synonymized to *E. cobayae* [14] or to *E. muris* [172]
*E. citelli* [239]	squirrels	Synonymized to *E. muris* [237]
*E. cobayae* [240]	guinea pigs	Nomen nudum [73] or synonymized to *E. muris* [172]
*E. criceti* [241]	hamsters	Synonymized to *E. muris* [237]
*E. decumani* [60]	mice and rats	Synonymized to *E. muris* [237]
*E. dipodomoysi* [242]	kangaroo rats	Synonymized to *E. muris* [237]
*E. marmotae* [243]	woodchucks	Synonymized to *E. muris* [237]
*E. muris* [21]	mice	
*E. ratti* [60]	rats	Synonymized to *E. muris* [237]
*E. bradypi* [157]	sloths	
*E. gallinarum* [244]	poultry	
*E. lacerticoli* [245]	lizards	
*E. flaviviridis* [190]	geckos (lizards)	
*E. ctenosaurae* [179]	iguanas	

**Table 5:** One-nucleate cyst-forming *Entamoeba* species (bovis group) described since 1903. Some species were originally described under the genera *Amoeba* or *Endamoeba*. Species are grouped by host category and ordered alphabetically. NHPs—non-human primates.

Original Species Epithet	Type Host (Other Hosts)	Comments
*E. chattoni* [158]	monkeys	
*E. polecki* [98]	pigs (humans, NHPs, ostriches, rheas)	
*E. antilocapra* [259]	antelopes	Synonymized to *E. bovis* [260]
*E. bovis* [261]	cattle (poligastric ruminants)	
*E. bubalus* [262]	water buffalo	
*E. debliecki* [263]	goats, pigs	Synonymized to *E. polecki* [102]
*E. dilimani* [264]	goats	
*E. ovis* [158]	sheep	Synonymized to *E. bovis* [202]
*E. suis* [265]	pigs (NHPs, ostriches)	Synonymized to *E. polecki* [14]; resurrected in 2006 [196]
*E. struthionis* [77]	ostriches (humans, pigs)	
*E. ilowaiskii* [219]	frogs	
*E. chiangraiensis* [90]	Freshwater eels	
*E. gadi* [233]	Marine fishes	

**Table 6:** Non cyst-forming *Entamoeba* species (gingivalis group) described since 1903. Species previously described by Gros [6] that are currently regarded as valid are also included. Species are grouped by host category and ordered alphabetically. NHPs—non-human primates.

Original Species Epithet	Type Host (Other Hosts)	Comments
*E. canibuccalis* [299]	dogs	Synonymized to *E. gingivalis* [73]
*E. gingivalis* [6]	humans (NHPs)	
*E. equibuccalis* [300]	horses	
*E. suigingivalis* [301]	Pigs	

**Table 7:** Insufficiently known *Entamoeba* species described since 1903. Species are ordered alphabetically.

Original Species Epithet	Type Host (Other Hosts)	Comments
*E. barreti* [230]	Turtles	
*E. bobaci* [302]	Marmots	Synonymized to *E. muris* [237]
*E. caprae* [303]	Goats	Nomen nudum [3]
*E. elephantis* [304]	Elephants	
*E. gedoelsti* [305]	Horses	Originally described as *Ameba intestinalis* Gedoelst 1911, the name was preoccupied by *A. intestinalis* Blanchard 1885 (synonym of *E. coli*); it was renamed *E. gedoelsti* [198]
*E. molae* [232]	marine fishes	
*E. pimelodi* [235]	freshwater fishes	
*E. varani* [306]	Nile monitor	

## Data Availability

No new data were created in this study.
